# Nonuniformity of P-values Can Occur Early in Diverging Dimensions

**Published:** 2019

**Authors:** Yingying Fan, Emre Demirkaya, Jinchi Lv

**Affiliations:** Data Sciences and Operations Department, University of Southern California, Los Angeles, CA 90089, USA; Business Analytics & Statistics, The University of Tennessee, Knoxville, Knoxville, TN 37996-4140, USA; Data Sciences and Operations Department, University of Southern California, Los Angeles, CA 90089, USA

**Keywords:** Nonuniformity, p-value, breakdown point, generalized linear model, high dimensionality, joint significance testing

## Abstract

Evaluating the joint significance of covariates is of fundamental importance in a wide range of applications. To this end, p-values are frequently employed and produced by algorithms that are powered by classical large-sample asymptotic theory. It is well known that the conventional p-values in Gaussian linear model are valid even when the dimensionality is a non-vanishing fraction of the sample size, but can break down when the design matrix becomes singular in higher dimensions or when the error distribution deviates from Gaussianity. A natural question is when the conventional p-values in generalized linear models become invalid in diverging dimensions. We establish that such a breakdown can occur early in nonlinear models. Our theoretical characterizations are confirmed by simulation studies.

## 1. Introduction

In many applications it is often desirable to evaluate the significance of covariates in a predictive model for some response of interest. Identifying a set of significant covariates can facilitate domain experts to further probe their causal relationships with the response. Ruling out insignificant covariates can also help reduce the fraction of false discoveries and narrow down the scope of follow-up experimental studies by scientists. These tasks certainly require an accurate measure of feature significance in finite samples. The tool of p-values has provided a powerful framework for such investigations.

As p-values are routinely produced by algorithms, practitioners should perhaps be aware that those p-values are usually based on classical large-sample asymptotic theory. For example, marginal p-values have been employed frequently in large-scale applications when the number of covariates *p* greatly exceeds the number of observations *n*. Those p-values are based on marginal regression models linking each individual covariate to the response separately. In these marginal regression models, the ratio of sample size to model dimensionality is equal to *n*, which results in justified p-values as sample size increases. Yet due to the correlations among the covariates, we often would like to investigate the joint significance of a covariate in a regression model conditional on all other covariates, which is the main focus of this paper. A natural question is whether conventional joint p-values continue to be valid in the regime of diverging dimensionality *p*.

It is well known that fitting the linear regression model with *p* > *n* using the ordinary least squares can lead to perfect fit giving rise to zero residual vector, which renders the p-values undefined. When *p* ≥ *n* and the design matrix is nonsingular, the p-values in the linear regression model are well defined and valid thanks to the exact normality of the least-squares estimator when the random error is Gaussian and the design matrix is deterministic. When the error is non-Gaussian, Huber (1973) showed that the least-squares estimator can still be asymptotically normal under the assumption of *p* = *o*(*n*), but is generally no longer normal when *p* = *o*(*n*) fails to hold, making the conventional p-values inaccurate in higher dimensions. For the asymptotic properties of *M*-estimators for robust regression, see, for example, Huber (1973); Portnoy (1984, 1985) for the case of diverging dimensionality *p* = *o*(*n*) and Karoui et al. (2013); Bean et al. (2013) for the scenario when the dimensionality *p* grows proportionally to sample size *n*.

We have seen that the conventional p-values for the least-squares estimator in linear regression model can start behaving wildly and become invalid when the dimensionality *p* is of the same order as sample size *n* and the error distribution deviates from Gaussianity. A natural question is whether similar phenomenon holds for the conventional p-values for the maximum likelihood estimator (MLE) in the setting of diverging-dimensional nonlinear models. More specifically, we aim to answer the question of whether *p* ~ *n* is still the breakdown point of the conventional p-values when we move away from the regime of linear regression model, where ~ stands for asymptotic order. To simplify the technical presentation, in this paper we adopt the generalized linear model (GLM) as a specific family of nonlinear models (McCullagh and Nelder, 1989). The GLM with a canonical link assumes that the conditional distribution of **y** given **X** belongs to the canonical exponential family, having the following density function with respect to some fixed measure

(1) $$f_{n}(y;X,\beta )\;\equiv \;\prod_{i=1}^{n}f_{0}\left(y_{i};\theta_{i}\right)\;=\;\prod_{i=1}^{n}\left\{c\left(y_{i}\right)\text{exp}\left[\frac{y_{i}\theta_{i}-b\left(\theta_{i}\right)}{\phi }\right]\right\},$$

where **X** = (**x**_1_, ⋯ , **x**_*p*_) is an *n* × *p* design matrix with **x**_*j*_ = (*x*_1*j*_, ⋯ , *x*_*nj*_)^*T*^, *j* = 1, ⋯ , *p*, **y** = (*y*_1_, ⋯ , *y*_*n*_)^*T*^ is an *n*-dimensional response vector, ***β*** = (*β*_1_, ⋯ , *β*_*p*_)^*T*^ is a *p*-dimensional regression coefficient vector, $\left\{f_{0}(y;\theta )\;:\;\theta \in \mathbb{R}\right\}$ is a family of distributions in the regular exponential family with dispersion parameter *ϕ* ∈ (0, ∞), and ***θ*** = (*θ*_1_, ⋯ , *θ*_*n*_)^*T*^ = **X*β***. As is common in GLM, the function *b*(*θ*) in (1) is implicitly assumed to be twice continuously differentiable with b″ (*θ*) always positive. Popularly used GLMs include the linear regression model, logistic regression model, and Poisson regression model for continuous, binary, and count data of responses, respectively.

The key innovation of our paper is the formal justification that the conventional p-values in nonlinear models of GLMs can become invalid in diverging dimensions and such a breakdown can occur *much earlier* than in linear models, which spells out a fundamental difference between linear models and nonlinear models. To begin investigating p-values in diverging-dimensional GLMs, let us gain some insights into this problem by looking at the specific case of logistic regression. Recently, Candès (2016) established an interesting phase transition phenomenon of perfect hyperplane separation for high-dimensional classification with an elegant probabilistic argument. Suppose we are given a random design matrix **X** ~ *N*(**0**, *I*_*n*_ ⊗ *I*_*p*_) and arbitrary binary *y*_*i*_’s that are not all the same. The phase transition of perfect hyperplane separation happens at the point *p*/*n* = 1/2. With such a separating hyperplane, there exist some $\beta^{*}\in {\mathbb{R}}^{p}$ and t∈ℝ such that $x_{i}^{T}\beta^{*}\;>\;t$ for all cases *y*_*i*_ = 1 and $x_{i}^{T}\beta^{*}\;<\;t$ for all controls *y*_*i*_ = 0. Let us fit a logistic regression model with an intercept. It is easy to show that multiplying the vector (−*t*, (β*)^*T*^)^*T*^ by a divergence sequence of positive numbers *c*, we can obtain a sequence of logistic regression fits with the fitted response vector approaching **y** = (*y*_1_, ⋯ , *y*_*n*_)^*T*^ as *c* → ∞. As a consequence, the MLE algorithm can return a pretty wild estimate that is close to infinity in topology when the algorithm is set to stop. Clearly, in such a case the p-value of the MLE is no longer justified and meaningful. The results in Candès (2016) have two important implications. First, such results reveal that unlike in linear models, p-values in nonlinear models can break down and behave wildly when *p*/*n* is of order 1/2; see Karoui et al. (2013); Bean et al. (2013) and discussions below. Second, these results motivate us to characterize the breakdown point of p-values in nonlinear GLMs with $p\sim n^{\alpha_{0}}$ in the regime of *α*_0_ ∈ [0, 1/2). In fact, our results show that the breakdown point can be even much earlier than *n*/2.

It is worth mentioning that our work is different in goals from the limited but growing literature on p-values for high-dimensional nonlinear models, and makes novel contributions to such a problem. The key distinction is that existing work has focused primarily on identifying the scenarios in which conventional p-values or their modifications continue to be valid with some sparsity assumption limiting the growth of intrinsic dimensions. For example, Fan and Peng (2004) established the oracle property including the asymptotic normality for nonconcave penalized likelihood estimators in the scenario of *p* = *o*(*n*^1/5^), while Fan and Lv (2011) extended their results to the GLM setting of non-polynomial (NP) dimensionality. In the latter work, the p-values were proved to be valid under the assumption that the intrinsic dimensionality *s* = *o*(*n*^1/3^). More recent work on high-dimensional inference in nonlinear model settings includes van de Geer et al. (2014); Athey et al. (2016) under sparsity assumptions. In addition, two tests were introduced in Guo and Chen (2016) for high-dimensional GLMs without or with nuisance regression parameters, but the p-values were obtained for testing the global hypothesis for a given set of covariates, which is different from our goal of testing the significance of individual covariates simultaneously. Portnoy (1988) studied the asymptotic behavior of the MLE for exponential families under the classical i.i.d. non-regression setting, but with diverging dimensionality. In contrast, our work under the GLM assumes the regression setting in which the design matrix **X** plays an important role in the asymptotic behavior of the MLE $\hat{\beta }$. The validity of the asymptotic normality of the MLE was established in Portnoy (1988) under the condition of *p* = *o*(*n*^1/2^), but the precise breakdown point in diverging dimensionality was not investigated therein. Another line of work is focused on generating asymptotically valid p-values when *p*/*n* converges to a fixed positive constant. For instance, Karoui et al. (2013) and Bean et al. (2013) considered *M*-estimators in the linear model and showed that their variance is greater than classically predicted. Based on this result, it is possible to produce p-values by making adjustments for the inflated variance in high dimensions. Recently, Sur and Candès (2018) showed that similar adjustment is possible for the likelihood ratio test (LRT) for logistic regression. Our work differs from this line of work in two important aspects. First, our focus is on the *classical* p-values and their validity. Second, their results concern dimensionality that is comparable to sample size, while we aim to analyze the problem for a lower range of dimensionality and pinpoint the exact breakdown point of p-values.

The rest of the paper is organized as follows. Section 2 provides characterizations of p-values in low dimensions. We establish the nonuniformity of GLM p-values in diverging dimensions in Section 3. Section 4 presents several simulation examples verifying the theoretical phenomenon. We discuss some implications of our results in Section 5. The proofs of all the results are relegated to the Appendix.

## 2. Characterizations of P-values in Low Dimensions

To pinpoint the breakdown point of GLM p-values in diverging dimensions, we start with characterizing p-values in low dimensions. In contrast to existing work on the asymptotic distribution of the penalized MLE, our results in this section focus on the asymptotic normality of the unpenalized MLE in diverging-dimensional GLMs, which justifies the validity of conventional p-values. Although Theorems 1 and 4 to be presented in Sections 2.2 and A are in the conventional sense of relatively small *p*, to the best of our knowledge such results are not available in the literature before in terms of the maximum range of dimensionality *p* without any sparsity assumption.

### 2.1. Maximum likelihood estimation

For the GLM (1), the log-likelihood log *f*_*n*_(**y**; **X**, *β*) of the sample is given, up to an affine transformation, by

(2) $$l_{n}(\beta )\;=\;n^{-1}\left[y^{T}X\beta -1^{T}b(X\beta )\right],$$

where **b**(***θ***) = (*b*(*θ*_1_), ⋯ , *b*(*θ*_*n*_))^*T*^ for $\theta \;=\;{\left(\theta_{1},\;\cdots \;,\theta_{n}\right)}^{T}\;\in {\mathbb{R}}^{n}$. Denote by $\hat{\beta }\;=\;{\left({\hat{\beta }}_{1},\;\cdots \;,{\hat{\beta }}_{p}\right)}^{T}\;\in \;{\mathbb{R}}^{p}$ the MLE which is the maximizer of (2), and

(3) $$\mu (\theta )\;=\;{\left(b^{\prime }\left(\theta_{1}\right),\;\cdots \;,b^{\prime }\left(\theta_{n}\right)\right)}^{T}\text{ and }\Sigma (\theta )\;=\;\text{diag}\left\{b^{\prime \prime }\left(\theta_{1}\right),\;\cdots \;,b^{\prime \prime }\left(\theta_{n}\right)\right\}.$$

A well-known fact is that the *n*-dimensional response vector **y** in GLM (1) has mean vector ***μ***(***θ***) and covariance matrix *ϕ***Σ**(***θ***). Clearly, the MLE $\hat{\beta }$ is given by the unique solution to the score equation

(4) $$X^{T}[y-\mu (X\beta )]\;=\;0$$

when the design matrix **X** is of full column rank *p*.

It is worth mentioning that for the linear model, the score equation (4) becomes the well-known normal equation **X**^*T*^**y** = **X**^*T*^**X*β*** which admits a closed form solution. On the other hand, equation (4) does not admit a closed form solution in general nonlinear models. This fact due to the nonlinearity of the mean function ***μ***(·) causes the key diffierence between the linear and nonlinear models. In future presentations, we will occasionally use the term *nonlinear GLMs* to exclude the linear model from the family of GLMs when necessary.

We will present in the next two sections some sufficient conditions under which the asymptotic normality of MLE holds. In particular, Section 2.2 concerns the case of fixed design and Section A deals with the case of random design. In addition, Section 2.2 allows for general regression coefficient vector ***β***_0_ and the results extend some existing ones in the literature, while Section A assumes the global null ***β***_0_ = **0** and Gaussian random design which enable us to pinpoint the exact breakdown point of the asymptotic normality for the MLE.

### 2.2. Conventional p-values in low dimensions under fixed design

Recall that we condition on the design matrix **X** in this section. We first introduce a deviation probability bound that facilitates our technical analysis. Consider both cases of bounded responses and unbounded responses. In the latter case, assume that there exist some constants *M*, *υ*_0_ > 0 such that

(5) $$\underset{1\leq i\leq n}{\text{max}}\;E\left\{\text{exp}\left[\frac{\left|y_{i}-b^{\prime }\left(\theta_{0,i}\right)\right|}{M}\right]-1-\frac{\left|y_{i}-b^{\prime }\left(\theta_{0,i}\right)\right|}{M}\right\}M^{2}\;\leq \;\frac{v_{0}}{2}$$

with (***θ***_0,1_, ⋯ , *θ*_0,*n*_)^*T*^ = ***θ***_0_ = **X*β***_0_, where ***β***_0_ = (***β***_0,1_, ⋯ , *β*_0,*p*_)^*T*^ denotes the true regression coefficient vector in model (1). Then by Fan and Lv (2011, 2013), it holds that for any $a\;\in \;{\mathbb{R}}^{n}$,

(6) $$P\left(\left|a^{T}Y-a^{T}\mu \left(\theta_{0}\right)\right|\;>\;\|a{\|}_{2}\varepsilon \right)\;\leq \;\varphi (\varepsilon ),$$

where $\varphi (\varepsilon )\;=\;2e^{-c_{1}\varepsilon^{2}}$ with *c*_1_ > 0 some constant, and *ε* ∈ (0, ∞) if the responses are bounded and *ε* ∈ (0, ‖**a**‖_2_/‖**a**‖_∞_] if the responses are unbounded.

For nonlinear GLMs, the MLE $\hat{\beta }$ solves the nonlinear score equation (4) whose solution generally does not admit an explicit form. To address such a challenge, we construct a solution to equation (4) in an asymptotically shrinking neighborhood of ***β***_0_ that meets the MLE $\hat{\beta }$ thanks to the uniqueness of the solution. Specifically, define a neighborhood of ***β***_0_ as

(7) $$N_{0}\;=\;\left\{\beta \in {\mathbb{R}}^{p}:{\left\|\beta -\beta_{0}\right\|}_{\infty }\;\leq \;n^{-\gamma }\;\text{log}\;n\right\}$$

for some constant *γ* ∈ (0, 1/2]. Assume that $p\;=\;O\left(n^{\alpha_{0}}\right)$ for some *α*_0_ ∈ (0, γ) and let $b_{n}\;=\;o\left\{\text{min}(n^{1/2-\gamma }\sqrt{\text{log}\;n},s_{n}^{-1}n^{2\gamma -\alpha_{0}-1/2}/{\left(\text{log}\;n\right)}^{2}\right\}$ be a diverging sequence of positive numbers, where *s*_*n*_ is a sequence of positive numbers that will be specified in heorem 1 below. We need some basic regularity conditions to establish the asymptotic normality of the MLE $\hat{\beta }$.

#### Condition 1

The design matrix **X** satisfies

(8) $${\left\|{\left[X^{T}\Sigma \left(\theta_{0}\right)X\right]}^{-1}\right\|}_{\infty }\;=\;O\left(b_{n}n^{-1}\right),$$

(9) $$\underset{\beta \in N_{0}}{\text{max}}\;{\text{max}}_{j=1}^{p}\;\lambda_{\text{max}}\left[X^{T}\text{diag}\left\{\left|x_{j}\right|\circ \left|\mu^{\prime \prime }(X\beta )\right|\right\}X\right]\;=\;O(n)$$

with ∘ denoting the Hadamard product and derivatives understood componentwise. Assume that ${\text{max}}_{j=1}^{p}{\left\|x_{j}\right\|}_{\infty }<c_{1}^{1/2}{\left\{n/(\text{log}\;n)\right\}}^{1/2}$ if the responses are unbounded.

#### Condition 2

The eigenvalues of *n*^−1^**A**_*n*_ are bounded away from 0 and ∞, $\sum_{i=1}^{n}{\left(z_{i}^{T}A_{n}^{-1}z_{i}\right)}^{3/2}=o\left(1\right)$, and ${\text{max}}_{i=1}^{n}\;E{\left|y_{i}-b^{\prime }\left(\theta_{0,i}\right)\right|}^{3}=O(1)$, where **A**_*n*_ = **X**^*T*^**Σ**(***θ***_**0**_)**X** and (**z**_1_, ⋯ , **z**_*n*_)^*T*^ = **X**.

Conditions 1 and 2 put some basic restrictions on the design matrix **X** and a moment condition on the responses. For the case of linear model, bound (8) becomes ‖(**X**^*T*^**X**)^−1^‖∞ = *O*(*b*_*n*_/*n*) and bound (9) holds automatically since *b′′′* (*θ*) ≡ 0. Condition 2 is related to the Lyapunov condition.

#### Theorem 1 (Asymptotic normality)

Assume that Conditions 1–2 and probability bound (6) hold. Then

- there exists a unique solution $\hat{\beta }$ to score equation (4) in $N_{0}$ with asymptotic probability one;
- the MLE $\hat{\beta }$ satisfies that for each vector $u\;\in \;{\mathbb{R}}^{p}$ with ‖**u**‖_2_ = 1 and ‖**u**‖_1_ = *O*(*s*_*n*_),
(10) $${\left(u^{T}A_{n}^{-1}u\right)}^{-1/2}\left(u^{T}\hat{\beta }-u^{T}\beta_{0}\right)\overset{D}{\to }N(0,\phi )$$
  and specifically for each 1 ≤ *j* ≤ *p*,
(11) $${\left(A_{n}^{-1}\right)}_{jj}^{-1/2}\left({\hat{\beta }}_{j}-\beta_{0,j}\right)\overset{D}{\to }N(0,\phi ),$$
  where **A**_*n*_ = **X**^*T*^**Σ**(***θ***_0_)**X** and ${\left(A_{n}^{-1}\right)}_{jj}$ denotes the jth diagonal entry of matrix $A_{n}^{-1}$.

Theorem 1 establishes the asymptotic normality of the MLE and consequently justifies the validity of the conventional p-values in low dimensions. Note that for simplicity, we present here only the marginal asymptotic normality, and the joint asymptotic normality also holds for the projection of the MLE onto any fixed-dimensional subspace. This result can also be extended to the case of misspecified models; see, for example, Lv and Liu (2014).

As mentioned in the Introduction, the asymptotic normality was shown in Fan and Lv (2011) for nonconcave penalized MLE having intrinsic dimensionality *s* = *o*(*n*^1/3^). In contrast, our result in Theorem 1 allows for the scenario of *p* = *o*(*n*^1/2^) with no sparsity assumption in view of our technical conditions. In particular, we see that the conventional p-values in GLMs generally remain valid in the regime of slowly diverging dimensionality *p* = *o*(*n*^1/2^).

## 3. Nonuniformity of GLM P-values in Diverging Dimensions

So far we have seen that for nonlinear GLMs, the p-values can be valid when *p* = *o*(*n*^1/2^) as shown in Section 2, and can become meaningless when *p* ≥ *n*/2 as discussed in the Introduction. Apparently, there is a big gap between these two regimes of growth of dimensionality *p*. To provide some guidance on the practical use of p-values in nonlinear GLMs, it is of crucial importance to characterize their breakdown point. To highlight the main message with simplified technical presentation, hereafter we content ourselves with the specific case of logistic regression model for binary response. Moreover, we investigate the distributional property in (11) for the scenario of true regression coefficient vector ***β***_0_ = **0**, that is, under the global null. We argue that this specific model is sufficient for our purpose because if the conventional p-values derived from MLEs fail (i.e., (11) fails) for at least one ***β***_0_ (in particular ***β***_0_ = **0**), then conventional p-values are not justified. Therefore, the breakdown point for logistic regression is at least the breakdown point for general nonlinear GLMs. This argument is fundamentally different from that of proving the overall validity of conventional p-values, where one needs to prove the asymptotic normality of MLEs under general GLMs rather than any specific model.

### 3.1. The wild side of nonlinear regime

For the logistic regression model (1), we have *b*(*θ*) = log(1 + e^*θ*^), θ∈ℝ and *ϕ* = 1. The mean vector ***μ***(***θ***) and covariance matrix *ϕ***Σ**(***θ***) of the *n*-dimensional response vector **y** given by (3) now take the familiar form of $\mu (\theta )\;=\;{\left(\frac{e^{\theta_{1}}}{1+e^{\theta_{1}}},\;\cdots \;,\frac{e^{\theta_{n}}}{1+e^{\theta_{n}}}\right)}^{T}$ and

$$\Sigma (\theta )\;=\;\text{diag}\left\{\frac{e^{\theta_{1}}}{{\left(1+e^{\theta_{1}}\right)}^{2}},\;\cdots \;,\frac{e^{\theta_{n}}}{{\left(1+e^{\theta_{n}}\right)}^{2}}\right\}$$

with ***θ*** = (*θ*_1_, ⋯ , *θ*_*n*_)^*T*^ = **X*β***. In many real applications, one would like to interpret the significance of each individual covariate produced by algorithms based on the conventional asymptotic normality of the MLE as established in Theorem 1. As argued at the beginning of this section, in order to justify the validity of p-values in GLMs, the underlying theory should at least ensure that the distributional property (11) holds for logistic regression under the global null. As we will see empirically in Section 4, as the dimensionality increases, p-values from logistic regression under the global null have a distribution that is skewed more and more toward zero. Consequently, classical hypothesis testing methods which reject the null hypothesis when p-value is less than the pre-specified level *α* would result in more false discoveries than the desired level of *α*. As a result, practitioners may simply lose the theoretical backup and the resulting decisions based on the p-values can become ineffective or even misleading. For this reason, it is important and helpful to identify the breakdown point of p-values in diverging-dimensional logistic regression model under the global null.

Characterizing the breakdown point of p-values in nonlinear GLMs is highly nontrivial and challenging. First, the nonlinearity generally causes the MLE to take no analytical form, which makes it di cult to analyze its behavior in diverging dimensions. Second, conventional probabilistic arguments for establishing the central limit theorem of MLE only enable us to see when the distributional property holds, but not exactly at what point it fails. To address these important challenges, we introduce novel geometric and probabilistic arguments presented later in the proofs of Theorems 2–3 that provide a rather delicate analysis of the MLE. In particular, our arguments unveil that the early breakdown point of p-values in nonlinear GLMs is essentially due to the *nonlinearity* of the mean function ***μ***(·). This shows that p-values can behave wildly much early on in diverging dimensions when we move away from linear regression model to nonlinear regression models such as the widely applied logistic regression; see the Introduction for detailed discussions on the p-values in diverging-dimensional linear models.

Before presenting the main results, let us look at the specific case of logistic regression model under the global null. In such a scenario, it holds that ***θ***_0_ = **X*β***_0_ = **0** and thus **Σ**(***θ***_0_) = 4^−1^*I*_*n*_, which results in

$$A_{n}\;=\;X^{T}\Sigma \left(\theta_{0}\right)X\;=\;4^{-1}X^{T}X.$$

In particular, we see that when *n*^−1^**X**^*T*^**X** is close to the identity matrix *I*_*p*_, the asymptotic standard deviation of the *j*th component ${\hat{\beta }}_{j}$ of the MLE $\hat{\beta }$ is close 2*n*^−1/2^ when the asymptotic theory in (11) holds. As mentioned in the Introduction, when *p* ≥ *n*/2 the MLE can blow up with excessively large variance, a strong evidence against the distributional property in (11). In fact, one can also observe inflated variance of the MLE relative to what is predicted by the asymptotic theory in (11) even when the dimensionality *p* grows at a slower rate with sample size *n*. As a consequence, the conventional p-values given by algorithms according to property (11) can be much biased toward zero and thus produce more significant discoveries than the truth. Such a breakdown of conventional p-values is delineated clearly in the simulation examples presented in Section 4.

### 3.2. Main results

We now present the formal results on the invalidity of GLM p-values in diverging dimensions.

#### Theorem 2 (Uniform orthonormal design)^1^

Assume that *n*^−1/2^**X** is uniformly distributed on the Stiefel manifold $V_{p}\left({\mathbb{R}}^{n}\right)$ consisting of all *n* × *p* orthonormal matrices. Then for the logistic regression model under the global null, the asymptotic normality of the MLE established in (11) fails to hold when *p* ~ *n*^2/3^, where ~ stands for asymptotic order.

#### Theorem 3 (Correlated Gaussian design)

Assume that **X** ~ *N*(**0**, *I*_*n*_ ⊗ **Σ**) with covariance matrix **Σ** nonsingular. Then for the logistic regression model under the global null, the same conclusion as in Theorem 2 holds.

Theorem 4 in Appendix A states that under the global null in GLM with Gaussian design, the p-value based on the MLE remains valid as long as the dimensionality *p* diverges with *n* at a slower rate than *n*^2/3^. This together with Theorems 2 and 3 shows that under the global null, the exact breakdown point for the uniformity of p-value is *n*^2/3^. We acknowledge that these results are mainly for theoretical interests because in practice one cannot check precisely whether the global null assumption holds or not. However, these results clearly suggest that in GLM with diverging dimensionality, one needs to be very cautious when using p-values based on the MLE.

The key ingredients of our new geometric and probabilistic arguments are demonstrated in the proof of Theorem 2 in Section B.3. The assumption that the rescaled random design matrix *n*^−1/2^**X** has the Haar measure on the Stiefel manifold $V_{p}\left({\mathbb{R}}^{n}\right)$ greatly facilitates our technical analysis. The major theoretical finding is that the nonlinearity of the mean function ***μ***(·) can be negligible in determining the asymptotic distribution of MLE as given in (11) when the dimensionality *p* grows at a slower rate than *n*^2/3^, but such nonlinearity can become dominant and deform the conventional asymptotic normality when *p* grows at rate *n*^2/3^ or faster. See the last paragraph of Section B.3 for more detailed in-depth discussions on such an interesting phenomenon. Furthermore, the global null assumption is a crucial component of our geometric and probabilistic argument. The global null assumption along with the distributional assumption on the design matrix ensures the symmetry property of the MLE and the useful fact that the MLE can be asymptotically independent of the random design matrix. In the absence of such an assumption, we may suspect that p-values of the noise variables can be affected by the signal variables due to asymmetry. Indeed, our simulation study in Section 4 reveals that as the number of signal variables increases, the breakdown point of the p-values occurs even earlier.

Theorem 3 further establishes that the invalidity of GLM p-values in high dimensions beyond the scenario of orthonormal design matrices considered in Theorem 2. The breakdown of the conventional p-values occurs regardless of the correlation structure of the covariates.

Our theoretical derivations detailed in the Appendix also suggest that the conventional p-values in nonlinear GLMs can generally fail to be valid when $p\sim n^{\alpha_{0}}$ with *α*_0_ ranging between 1/2 and 2/3, which differs significantly from the phenomenon for linear models as discussed in the Introduction. The special feature of logistic regression model that the variance function *b″* (*θ*) takes the maximum value 1/4 at natural parameter *θ* = 0 leads to a higher transition point of $p\sim n^{\alpha_{0}}$ with *α*_0_ = 2/3 for the case of global null ***β***_0_ = **0**.

## 4. Numerical Studies

We now investigate the breakdown point of p-values for nonlinear GLMs in diverging dimensions as predicted by our major theoretical results in Section 3 with several simulation examples. Indeed, these theoretical results are well supported by the numerical studies.

### 4.1. Simulation examples

Following Theorems 2–3 in Section 3, we consider three examples of the logistic regression model (1). The response vector **y** = (*y*_1_, ⋯ , *y*_*n*_)^*T*^ has independent components and each *y*_*i*_ has Bernoulli distribution with parameter $e^{\theta_{i}}/\left(1\;+\;e^{\theta_{i}}\right)$, where ***θ*** = (*θ*_1_, ⋯ ,*θ*_*n*_)^*T*^ = **X*β***_0_. In example 1, we generate the *n* × *p* design matrix **X** = (**x**_**1**_, ⋯ , **x**_p_) such that *n*^−1/2^**X** is uniformly distributed on the Stiefel manifold $V_{p}\left({\mathbb{R}}^{n}\right)$ as in Theorem 2, while examples 2 and 3 assume that **X** ~ *N*(**0**, *I*_*n*_ ⊗ **Σ**) with covariance matrix **Σ** as in Theorem 3. In particular, we choose **Σ** = (*ρ*^|*j−k*|^)_1≤*j*,*k*≤*p*_ with *ρ* = 0, 0.5, and 0.8 to reflect low, moderate, and high correlation levels among the covariates. Moreover, examples 1 and 2 assume the global null model with ***β***_0_ = **0** following our theoretical results, whereas example 3 allows sparsity *s* = ‖***β***_0_‖_0_ to vary.

To examine the asymptotic results we set the sample size *n* = 1000. In each example, we consider a spectrum of dimensionality *p* with varying rate of growth with sample size *n*. As mentioned in the Introduction, the phase transition of perfect hyperplane separation happens at the point *p*/*n* = 1/2. Recall that Theorems 2–3 establish that the conventional GLM p-values can become invalid when *p* ~ *n*^2/3^. We set $p=\left[n^{\alpha_{0}}\right]$ with *α*_0_ in the grid {2/3 – 4*δ*, ⋯ , 2/3 – *δ*, 2/3, 2/3 + *δ*, ⋯ ,2/3 + 4*δ*, (log(*n*) – log(2))/log(*n*)} for *δ* = 0.05. For example 3, we pick *s* signals uniformly at random among all but the first components, where a random half of them are chosen as 3 and the other half are set as −3.

The goal of the simulation examples is to investigate empirically when the conventional GLM p-values could break down in diverging dimensions. When the asymptotic theory for the MLE in (11) holds, the conventional p-values would be valid and distributed uniformly on the interval [0, 1] under the null hypothesis. Note that the first covariate **x**_1_ is a null variable in each simulation example. Thus in each replication, we calculate the conventional *p*-value for testing the null hypothesis *H*_0_ : *β*_0,1_ = 0. To check the validity of these p-values, we further test their uniformity.

For each simulation example, we first calculate the p-values for a total of 1, 000 replications as described above and then test the uniformity of these 1, 000 p-values using, for example, the Kolmogorov–Smirnov (KS) test (Kolmogorov, 1933; Smirnov, 1948) and the Anderson–Darling (AD) test (Anderson and Darling, 1952, 1954). We repeat this procedure 100 times to obtain a final set of 100 new p-values from each of these two uniformity tests. Specifically, the KS and AD test statistics for testing the uniformity on [0, 1] are defined as

$$\text{KS}\;=\;\underset{x\in [0,1]}{\text{sup}}\left|F_{m}(x)-x\right|\text{ and AD}=\;m\int_{0}^{1}\frac{{\left[F_{m}(x)-x\right]}^{2}}{x(1-x)}dx,$$

respectively, where $F_{m}(x)\;=\;m^{-1}\sum_{i=1}^{m}I_{\left(-\infty ,x\right]}\left(x_{i}\right)$ is the empirical distribution function for a given sample ${\left\{x_{i}\right\}}_{i=1}^{m}$.

### 4.2. Testing results

**Figure 1:**
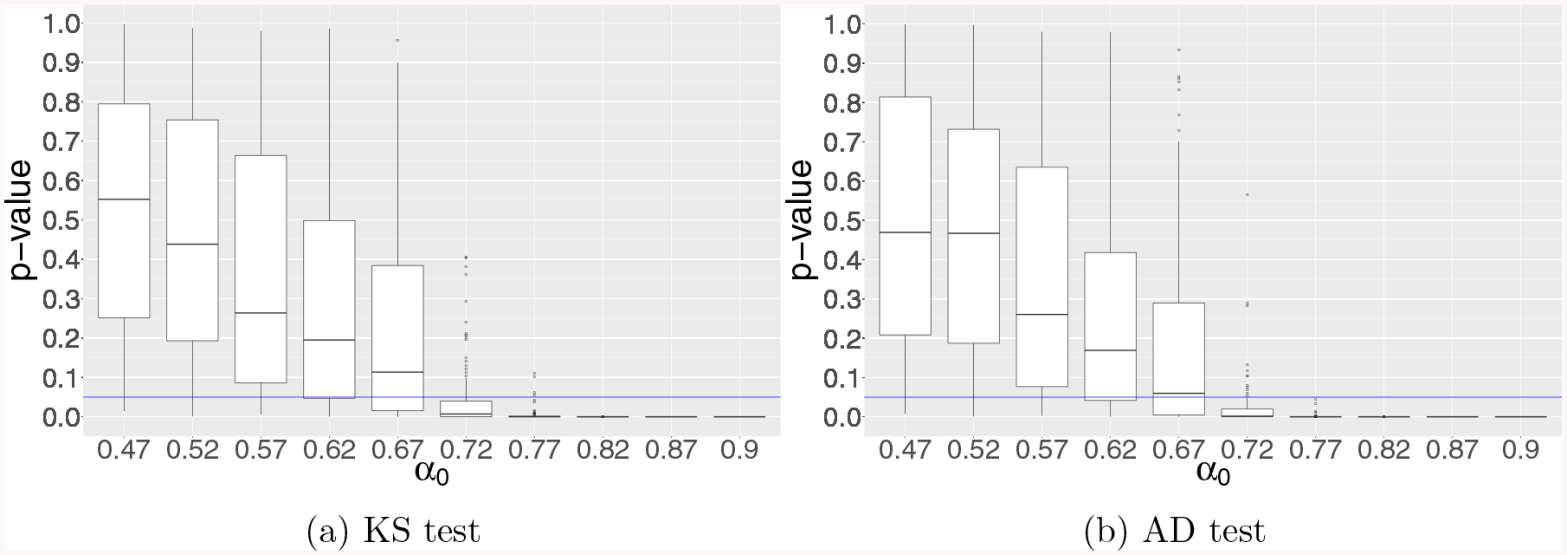
Results of KS and AD tests for testing the uniformity of GLM p-values in simulation example 1 for diverging-dimensional logistic regression model with uniform orthonormal design under global null. The vertical axis represents the p-value from the KS and AD tests, and the horizontal axis stands for the growth rate *α*_0_ of dimensionality *p* = [*n*^*α*0^].

**Figure 2:**
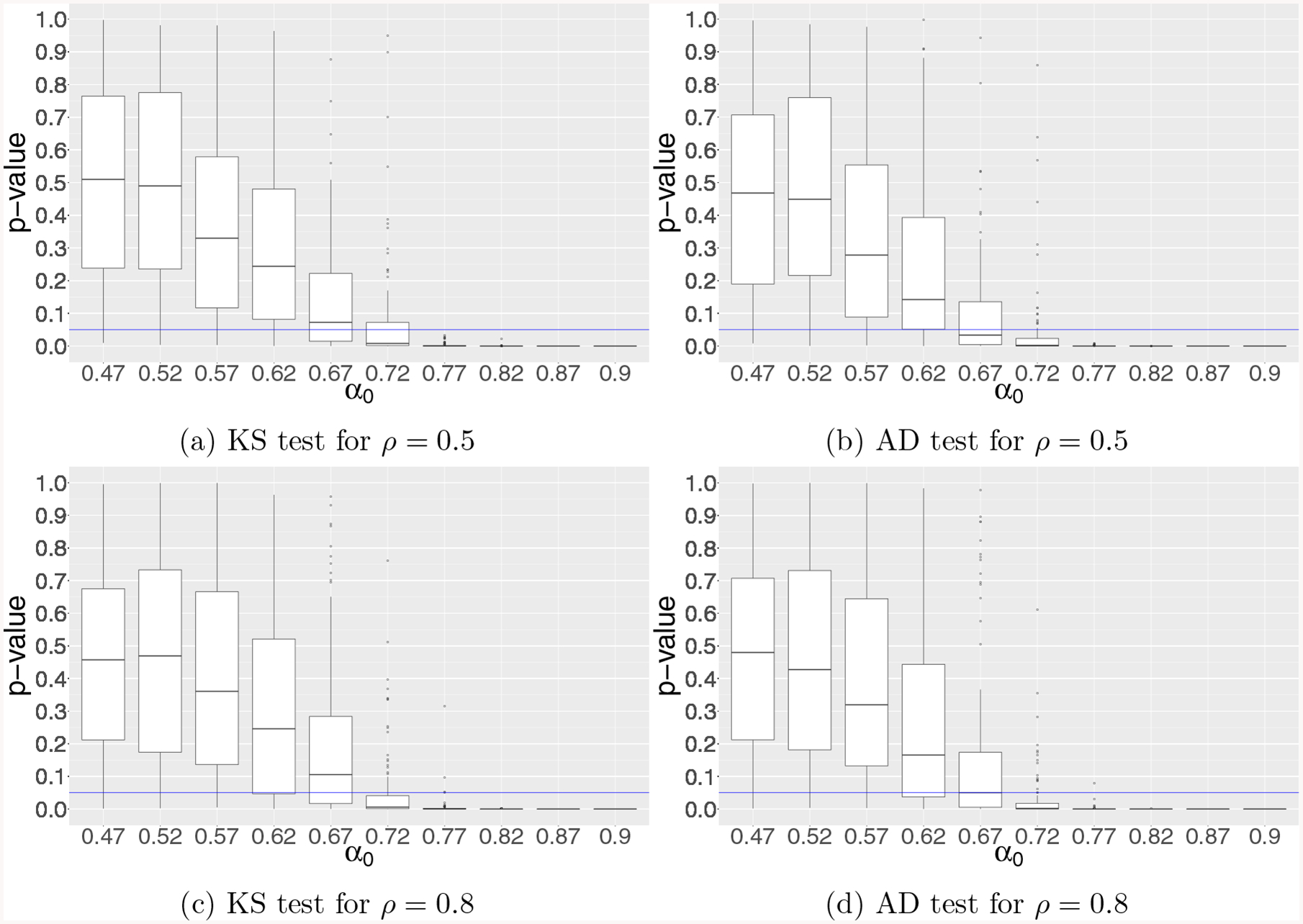
Results of KS and AD tests for testing the uniformity of GLM p-values in simulation example 2 for diverging-dimensional logistic regression model with correlated Gaussian design under global null for varying correlation level *ρ*. The vertical axis represents the p-value from the KS and AD tests, and the horizontal axis stands for the growth rate *α*_0_ of dimensionality *p* = [*n*^*α*0^].

**Figure 3:**
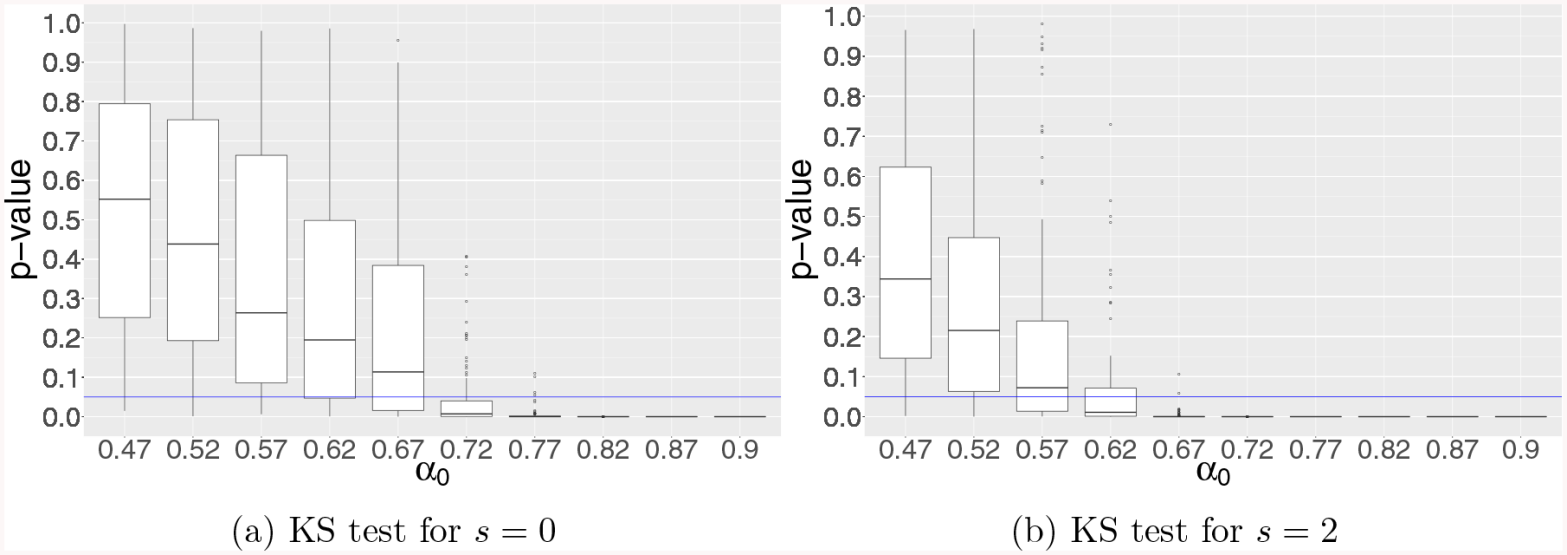
Results of KS test for testing the uniformity of GLM p-values in simulation example 3 for diverging-dimensional logistic regression model with uncorrelated Gaussian design under global null for varying sparsity *s*. The vertical axis represents the p-value from the KS test, and the horizontal axis stands for the growth rate *α*_0_ of dimensionality *p* = [*n*^*α*0^].

For each simulation example, we apply both KS and AD tests to verify the asymptotic theory for the MLE in (11) by testing the uniformity of conventional p-values at significance level 0.05. As mentioned in Section 4.1, we end up with two sets of 100 new p-values from the KS and AD tests. Figures 1–3 depict the boxplots of the p-values obtained from both KS and AD tests for simulation examples 1–3, respectively. In particular, we observe that the numerical results shown in Figures 1–2 for examples 1–2 are in line with our theoretical results established in Theorems 2–3, respectively, for diverging-dimensional logistic regression model under global null that the conventional p-values break down when $p\sim n^{\alpha_{0}}$ with *α*_0_ = 2/3. Figure 3 for example 3 examines the breakdown point of p-values with varying sparsity *s*. It is interesting to see that the breakdown point shifts even earlier when *s* increases as suggested in the discussions in Section 3.2. The results from the AD test are similar so we present only the results from the KS test for simplicity.

**Figure 4:**
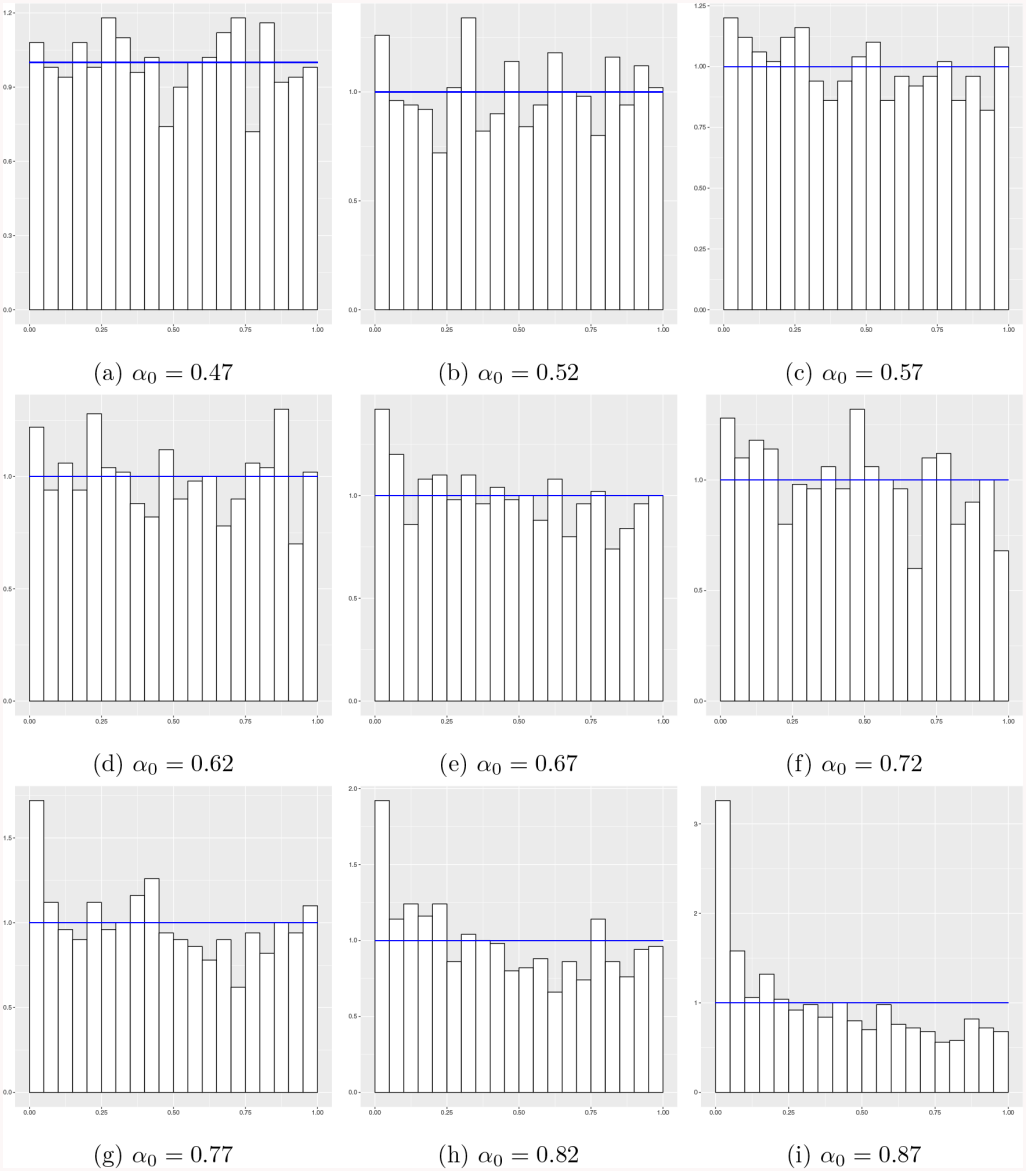
Histograms of null p-values in simulation example 1 from the first simulation repetition for different growth rates *α*_0_ of dimensionality *p* = [*n*^*α*0^].

To gain further insights into the nonuniformity of the null p-values, we next provide an additional figure in the setting of simulation example 1. Specifically, in Figure 4 we present the histograms of the 1,000 null p-values from the first simulation repetition (out of 100) for each value of *α*_0_. It is seen that as the dimensionality increases (i.e., *α*_0_ increases), the null p-values have a distribution that is skewed more and more toward zero, which is prone to produce more false discoveries if these p-values are used naively in classical hypothesis testing methods.

To further demonstrate the severity of the problem, we estimate the probability of making type I error at significance level *a*, as the fraction of p-values below *a*. The means and standard deviations of the estimated probabilities are reported in Table 1 for *a* = 0.05 and 0.1. When the null p-values are distributed uniformly, the probabilities of making type I error should all be close to the target level *a*. However, Table 1 shows that when the growth rate of dimensionality *α*_0_ approaches or exceeds 2/3, these probabilities can be much larger than *a*, which again supports our theoretical findings. Also it is seen that when *α*_0_ is close to but still smaller than 2/3, the averages of estimated probabilities exceed slightly *a*, which could be the effect of finite sample size.

## 5. Discussions

In this paper we have provided characterizations of p-values in nonlinear GLMs with diverging dimensionality. The major findings are that the conventional p-values can remain valid when *p* = *o*(*n*^1/2^), but can become invalid much earlier in nonlinear models than in linear models, where the latter case can allow for *p* = *o*(*n*). In particular, our theoretical results pinpoint the breakdown point of *p* ~ *n*^2/3^ for p-values in diverging-dimensional logistic regression model under global null with uniform orthonormal design and correlated Gaussian design, as evidenced in the numerical results. It would be interesting to investigate such a phenomenon for more general class of random design matrices.

The problem of identifying the breakdown point of p-values becomes even more complicated and challenging when we move away from the setting of global null. Our technical analysis suggests that the breakdown point $p\sim n^{\alpha_{0}}$ can shift even earlier with *α*_0_ ranging between 1/2 and 2/3. But the exact breakdown point can depend upon the number of signals *s*, the signal magnitude, and the correlation structure among the covariates in a rather complicated fashion. Thus more delicate mathematical analysis is needed to obtain the exact relationship. We leave such a problem for future investigation. Moving beyond the GLM setting will further complicate the theoretical analysis.

As we routinely produce p-values using algorithms, the phenomenon of nonuniformity of p-values occurring early in diverging dimensions unveiled in the paper poses useful cautions to researchers and practitioners when making decisions in real applications using results from p-value based methods. For instance, when testing the joint significance of covariates in diverging-dimensional nonlinear models, the effective sample size requirement should be checked before interpreting the testing results. Indeed, statistical inference in general high-dimensional nonlinear models is particularly challenging since obtaining accurate p-values is generally not easy. One possible route is to bypass the use of p-values in certain tasks including the false discovery rate (FDR) control; see, for example, Barber and Candès (2015); Candès et al. (2018); Fan et al. (2018) for some initial efforts made along this line.

## Figures and Tables

**Table 1:** Means and standard deviations (SD) for estimated probabilities of making type I error in simulation example 1 with *α*_0_ the growth rate of dimensionality *p* = [*n*^*α*0^]. Two significance levels *a* = 0.05 and 0.1 are considered.

	*α*_0_	0.10	0.47	0.57	0.67	0.77	0.87
*a* = 0.05	Mean	0.050	0.052	0.055	0.063	0.082	0.166
	SD	0.006	0.007	0.007	0.007	0.001	0.011
*a* = 0.1	Mean	0.098	0.104	0.107	0.118	0.144	0.247
	SD	0.008	0.010	0.009	0.011	0.012	0.013

## Footnotes

1. For completeness, we present Theorem 4 in Appendix A which provides a random design version of Theorem 1 under global null and a partial converse of Theorems 2 and 3.

## Appendix A. Conventional P-values in Low Dimensions under Random Design

Under the specific assumption of Gaussian design and global null ***β***_0_ = **0**, we can show that the asymptotic normality of MLE continues to hold without previous Conditions 1–2.

### Theorem 4

Assume that ***β***_0_ = **0**, the rows of **X** are i.i.d. from *N*(0, **Σ**)*, b*^(5)^(·) is uniformly bounded in its domain, and **y** − ***μ***_0_ has uniformly sub-Gaussian components. Then if *p* = *O*(*n*^*α*^) with some *α* ∈ [0, 2/3), we have the componentwise asymptotic normality

$${\left(A_{n}^{-1}\right)}_{jj}^{-1/2}{\hat{\beta }}_{j}\overset{D}{\to }N(0,\phi ),$$

where all the notation is the same as in (11).

Theorem 4 shows that the conclusions of Theorem 1 continue to hold for the case of random design and global null with the major difference that the dimensionality can be pushed as far as *p* ~ *n*^2/3^. The main reasons for presenting Theorem 4 under Gausssian design are twofold. First, Gaussian design is a widely used assumption in the literature. Second, our results on the nonuniformity of GLM p-values in diverging dimensions use geometric and probabilistic arguments which require random design setting; see Section 3 for more details. To contrast more accurately the two regimes and maintain self-contained theory, we have chosen to present Theorem 4 under Gaussian design. On the other hand, we would like to point out that Theorem 4 is not for practitioners who want to justify the usage of classical p-values. The global null assumption of ***β***_0_ = **0** restricts the validity of Theorem 4 in many practical scenarios.

## Appendix B. Proofs of Main Results

We provide the detailed proofs of Theorems 1–3 in this Appendix.

### B.1. Proof of Theorem 1

To ease the presentation, we split the proof into two parts, where the first part locates the MLE $\hat{\beta }$ in an asymptotically shrinking neighborhood $N_{0}$ of the true regression coefficient vector ***β***_0_ with significant probability and the second part further establishes its asymptotic normality.

**Part 1**: Existence of a unique solution to score equation (4) in $N_{0}$ under Condition 1 and probability bound (6). For simplicity, assume that the design matrix **X** is rescaled columnwise such that ${\left\|x_{j}\right\|}_{2}=\sqrt{n}$ for each 1 ≤ *j* ≤ *p*. Consider an event

(12)
$$E\;=\;\left\{\|\xi {\|}_{\infty }\leq c_{1}^{-1/2}\sqrt{n\;\text{log}\;n}\right\},$$

where ***ξ*** = (*ξ*_1_, ⋯ , ***ξ***_*p*_)^*T*^ = **X**^*T*^ [**y** − ***μ***(***θ***_0_)]. Note that for unbounded responses, the assumption of ${\text{max}}_{j=1}^{p}{\left\|x_{j}\right\|}_{\infty }<c_{1}^{1/2}{\left\{n/(\text{log}\;n)\right\}}^{1/2}$ in Condition 1 entails that $c_{1}^{-1/2}\sqrt{\text{log}\;n}<{\text{min}}_{j=1}^{p}\left\{{\left\|x_{j}\right\|}_{2}/{\left\|x_{j}\right\|}_{\infty }\right\}$. Thus by $\|x_{j}{\|}_{2}=\sqrt{n}$, probability bound (6), and Bonferroni’s inequality, we deduce

(13)
$$P(E)\;\geq \;1-\sum_{j=1}^{p}P\left(\left|\xi_{j}\right|>c_{1}^{-1/2}\sqrt{n\;\text{log}\;n}\right)\;\geq \;1\;-\;2pn^{-1}\;=\;1-O\left\{n^{-\left(1-\alpha_{0}\right)}\right\},$$

since $p=O\left(n^{\alpha_{0}}\right)$ for some *α*_0_ ∈ (0,*γ*) with *γ* ∈ (0, 1/2] by assumption. Hereafter we condition on the event E defined in (12) which holds with significant probability.

We will show that for sufficiently large *n*, the score equation (4) has a solution in the neighborhood $N_{0}$ which is a hypercube. Define two vector-valued functions

$$\gamma (\beta )\;=\;{\left(\gamma_{1}(\beta ),\;\cdots \;,\gamma_{p}(\beta )\right)}^{T}\;=\;X^{T}\mu (X\beta )$$

and

$$\Psi (\beta )\;=\;\gamma (\beta )-\gamma \left(\beta_{0}\right)-\xi ,\;\beta \in {\mathbb{R}}^{p}.$$

Then equation (4) is equivalent to **Ψ**(***β***) = **0**. We need to show that the latter has a solution inside the hypercube $N_{0}$. To this end, applying a second order Taylor expansion of ***γ***(***β***) around ***β***_0_ with the Lagrange remainder term componentwise leads to

(14)
$$\gamma (\beta )\;=\;\gamma \left(\beta_{0}\right)\;+\;X^{T}\Sigma \left(\theta_{0}\right)X\left(\beta -\beta_{0}\right)\;+\;r,$$

where **r** = (*r*_1_, ⋯ , *r*_*p*_)^*T*^ and for each 1 ≤ *j* ≤ *p*,

$$r_{j}\;=\;\frac{1}{2}{\left(\beta -\beta_{0}\right)}^{T}\nabla^{2}\gamma_{j}\left(\beta_{j}\right)\left(\beta -\beta_{0}\right)$$

with ***β***_*j*_ some *p*-dimensional vector lying on the line segment joining ***β*** and ***β***_0_. It follows from (9) in Condition 1 that

(15)
$${\left\|r\right\|}_{\infty }\leq \underset{\delta \in N_{0}}{\text{max}}\;\overset{p}{\underset{j=1}{\text{max}}}\frac{1}{2}\lambda_{\text{max}}\left[X^{T}\text{diag}\left\{\left|x_{j}\right|\circ \left|\mu^{\prime \prime }(X\delta )\right|\right\}X\right]{\left\|\beta -\beta_{0}\right\|}_{2}^{2}\;=O\left\{pn^{1-2\gamma }{\left(\text{log}\;n\right)}^{2}\right\}.$$

Let us define another vector-valued function

(16)
$$\bar{\Psi }(\beta )\;\equiv \;{\left[X^{T}\Sigma \left(\theta_{0}\right)X\right]}^{-1}\Psi (\beta )\;=\;\beta -\beta_{0}+u,$$

where **u** = −[**X**^*T*^**Σ**(***θ***_0_)**X**]^−1^(***ξ*** − **r**). It follows from (12), (15), and (8) in Condition 1 that for any $\beta \in N_{0}$,

(17)
$${\left\|u\right\|}_{\infty }\;\leq \;{\left\|{\left[X^{T}\Sigma \left(\theta_{0}\right)X\right]}^{-1}\right\|}_{\infty }\;\left(\|\xi {\|}_{\infty }+\|r{\|}_{\infty }\right)\;=O\left[b_{n}n^{-1/2}\sqrt{\;\text{log}\;n}+b_{n}pn^{-2\gamma }{\left(\text{log}\;n\right)}^{2}\right].$$

By the assumptions of $p\;=\;O\left(n^{\alpha_{0}}\right)$ with constant *α*_0_ ∈ (0, *γ*) and $b_{n}\;=\;o\left\{\text{min}(n^{1/2-\gamma }\sqrt{\text{log}\;n},\;n^{2\gamma -\alpha_{0}-1/2}/{\left(\text{log}\;n\right)}^{2}\right\}$, We have

$$\|u{\|}_{\infty }\;=\;o\left(n^{-\gamma }\;\text{log}\;n\right).$$

Thus in light of (16), it holds for large enough *n* that when ${\left(\beta -\beta_{0}\right)}_{j}\;=\;n^{-\gamma }\sqrt{\text{log}\;n}$,

(18)
$${\bar{\Psi }}_{j}\left(\beta \right)\;\geq \;n^{-\gamma }\;\sqrt{\text{log}\;n}-{\left\|u\right\|}_{\infty }\;\geq \;0,$$

and when ${\left(\beta -\beta_{0}\right)}_{j}\;=\;-n^{-\gamma }\sqrt{\text{log}\;n}$,

(19)
$${\bar{\Psi }}_{j}\left(\beta \right)\;\leq \;-n^{-\gamma }\sqrt{\text{log}\;n}+{\left\|u\right\|}_{\infty }\;\leq 0,$$

where $\bar{\Psi }(\beta )\;=\;{\left({\bar{\Psi }}_{1}(\beta ),\;\cdots \;,{\bar{\Psi }}_{p}(\beta )\right)}^{T}$.

By the continuity of the vector-valued function $\bar{\Psi }(\beta )$, (18), and (19), Miranda’s existence theorem Vrahatis (1989) ensures that equation $\bar{\Psi }(\beta )\;=\;0$ has a solution $\hat{\beta }$ in $N_{0}$. Clearly, $\hat{\beta }$ also solves equation Ψ(β)=0 in view of (16). Therefore, we have shown that score equation (4) indeed has a solution $\hat{\beta }$ in $N_{0}$. The strict concavity of the log-likelihood function (2) by assumptions for model (1) entails that $\hat{\beta }$ is the MLE.

**Part 2**: Conventional asymptotic normality of the MLE $\hat{\beta }$. Fix any 1 ≤ *j* ≤ *p*. In light of (16), we have $\hat{\beta }-\beta_{0}\;=\;A_{n}^{-1}(\xi \;-\;r)$, which results in

(20)
$${\left(A_{n}^{-1}\right)}_{jj}^{-1/2}\left({\hat{\beta }}_{j}-\beta_{0,j}\right)\;=\;{\left(A_{n}^{-1}\right)}_{jj}^{-1/2}e_{j}^{T}A_{n}^{-1}\xi -{\left(A_{n}^{-1}\right)}_{jj}^{-1/2}e_{j}^{T}A_{n}^{-1}r$$

with $e_{j}\in {\mathbb{R}}^{p}$ having one for the *j*th component and zero otherwise. Note that since the smallest and largest eigenvalues of *n*^−1^**A**_*n*_ are bounded away from 0 and ∞ by Condition 2, it is easy to show that ${\left(A_{n}^{-1}\right)}_{jj}^{-1/2}$ is of exact order *n*^1/2^. In view of (17), it holds on the event E defined in (12) that

$${\left\|A_{n}^{-1}r\right\|}_{\infty }\;\leq \;{\left\|{\left[X^{T}\Sigma \left(\theta_{0}\right)X\right]}^{-1}\right\|}_{\infty }\;{\left\|r\right\|}_{\infty }\;=\;O\left[b_{n}pn^{-2\gamma }{\left(\text{log}\;n\right)}^{2}\right]\;=\;o\left(n^{-1/2}\right),$$

since $b_{n}\;=\;o\left\{n^{2\gamma -\alpha_{0}-1/2}/{\left(\text{log}\;n\right)}^{2}\right\}$ by assumption. This leads to

(21)
$${\left(A_{n}^{-1}\right)}_{jj}^{-1/2}e_{j}^{T}A_{n}^{-1}r\;=\;O\left(n^{1/2}\right)\cdot o_{P}\left(n^{-1/2}\right)\;=\;o_{P}(1).$$

It remains to consider the term ${\left(A_{n}^{-1}\right)}_{jj}^{-1/2}e_{j}^{T}A_{n}^{-1}\xi \;=\;\sum_{i=1}^{n}\eta_{i}$, where $\eta_{i}\;=\;{\left(A_{n}^{-1}\right)}_{jj}^{-1/2}e_{j}^{T}A_{n}^{-1}z_{i}\left[y_{i}-b^{\prime }\left(\theta_{0,i}\right)\right]$. Clearly, the *n* random variables *η*_*i*_’s are independent with mean 0 and

$$\sum_{i=1}^{n}\text{var}\left(\eta_{i}\right)\;=\;{\left(A_{n}^{-1}\right)}_{jj}^{-1}e_{j}^{T}A_{n}^{-1}\left(\phi A_{n}\right)A_{n}^{-1}e_{j}\;=\;\phi .$$

It follows from Condition 2 and the Cauchy–Schwarz inequality that

$$\sum_{i=1}^{n}E{\left|\eta_{i}\right|}^{3}=\sum_{i=1}^{n}{\left|{\left(A_{n}^{-1}\right)}_{jj}^{-1/2}e_{j}^{T}A_{n}^{-1}z_{i}\right|}^{3}E{\left|y_{i}-b^{\prime }\left(\theta_{0,i}\right)\right|}^{3}\;=\;O(1)\;\sum_{i=1}^{n}{\left|{\left(A_{n}^{-1}\right)}_{jj}^{-1/2}e_{j}^{T}A_{n}^{-1}z_{i}\right|}^{3}\;\leq \;O(1)\;\sum_{i=1}^{n}{\left\|{\left(A_{n}^{-1}\right)}_{jj}^{-1/2}e_{j}^{T}A_{n}^{-1/2}\right\|}_{2}^{3}{\left\|A_{n}^{-1/2}z_{i}\right\|}_{2}^{3}\;=\;O(1)\;\sum_{i=1}^{n}{\left(z_{i}^{T}A_{n}^{-1}z_{i}\right)}^{3/2}\;=\;o(1).$$

Thus an application of Lyapunov’s theorem yields

(22)
$${\left(A_{n}^{-1}\right)}_{jj}^{-1/2}e_{j}^{T}A_{n}^{-1}\xi \;=\;\sum_{i=1}^{n}\eta_{i}\overset{D}{\to }N(0,\phi ).$$

By Slutsky’s lemma, we see from (20)–(22) that

$${\left(A_{n}^{-1}\right)}_{jj}^{-1/2}\left({\hat{\beta }}_{j}-\beta_{0,j}\right)\overset{D}{\to }N(0,\phi ),$$

showing the asymptotic normality of each component ${\hat{\beta }}_{j}$ of the MLE $\hat{\beta }$.

We further establish the asymptotic normality for the one-dimensional projections of the MLE $\hat{\beta }$. Fix an arbitrary vector $u\in {\mathbb{R}}^{p}$ with ‖**u**‖_2_ = 1 satisfying the *L*_1_ sparsity bound ‖**u**‖_1_ = *O*(*s*_*n*_). In light of (16), we have $\hat{\beta }-\beta_{0}\;=\;A_{n}^{-1}\left(\xi -r\right)$, which results in

(23)
$${\left(u^{T}A_{n}^{-1}u\right)}^{-1/2}\left(u^{T}\hat{\beta }-u^{T}\beta_{0}\right)\;=\;{\left(u^{T}A_{n}^{-1}u\right)}^{-1/2}u^{T}A_{n}^{-1}\xi -{\left(u^{T}A_{n}^{-1}u\right)}^{-1/2}u^{T}A_{n}^{-1}r.$$

Note that since the smallest and largest eigenvalues of *n*^−1^**A**_*n*_ are bounded away from 0 and ∞ by Condition 2, it is easy to show that ${\left(u^{T}A_{n}^{-1}u\right)}^{-1/2}$ is of exact order *n*^1/2^. In view of (17), it holds on the event E defined in (12) that

$${\left\|A_{n}^{-1}r\right\|}_{\infty }\;\leq \;{\left\|{\left[X^{T}\Sigma \left(\theta_{0}\right)X\right]}^{-1}\right\|}_{\infty }{\left\|r\right\|}_{\infty }\;=\;O\left\{b_{n}pn^{-2\gamma }{\left(\text{log}\;n\right)}^{2}\right\}\;=\;o\left(s_{n}^{-1}n^{-1/2}\right)$$

since $b_{n}\;=\;o\left\{s_{n}^{-1}n^{2\gamma -\alpha_{0}-1/2}/{\left(\text{log}\;n\right)}^{2}\right\}$ by assumption. This leads to

(24)
$${\left(u^{T}A_{n}^{-1}u\right)}^{-1/2}u^{T}A_{n}^{-1}r\;=\;O\left(n^{1/2}\right)\cdot {\left\|u\right\|}_{1}\cdot {\left\|A_{n}^{-1}r\right\|}_{\infty }\;=\;o_{P}(1)$$

since ‖**u**‖_1_ = *O*(*s*_*n*_) by assumption.

It remains to consider the term ${\left(u^{T}A_{n}^{-1}u\right)}^{-1/2}u^{T}A_{n}^{-1}\xi \;=\;\sum_{i=1}^{n}\eta_{i}$ with $\eta_{i}={\left(u^{T}A_{n}^{-1}u\right)}^{-1/2}u^{T}A_{n}^{-1}z_{i}\left[y_{i}-b^{\prime }\left(\theta_{0,i}\right)\right]$. Clearly, the *n* random variables *η*_*i*_’s are independent with mean 0 and

$$\sum_{i=1}^{n}\text{var}\left(\eta_{i}\right)\;=\;{\left(u^{T}A_{n}^{-1}u\right)}^{-1}u^{T}A_{n}^{-1}\left(\phi A_{n}\right)A_{n}^{-1}u\;=\;\phi .$$

It follows from Condition 2 and the Cauchy–Schwarz inequality that

$$\sum_{i=1}^{n}E{\left|\eta_{i}\right|}^{3}\;=\;\sum_{i=1}^{n}{\left|{\left(u^{T}A_{n}^{-1}u\right)}^{-1/2}u^{T}A_{n}^{-1}z_{i}\right|}^{3}E{\left|y_{i}-b^{\prime }\left(\theta_{0,i}\right)\right|}^{3}\;=\;O(1)\sum_{i=1}^{n}{\left|{\left(u^{T}A_{n}^{-1}u\right)}^{-1/2}u^{T}A_{n}^{-1}z_{i}\right|}^{3}\;\leq \;O(1)\;\sum_{i=1}^{n}{\left\|{\left(u^{T}A_{n}^{-1}u\right)}^{-1/2}u^{T}A_{n}^{-1/2}\right\|}_{2}^{3}{\left\|A_{n}^{-1/2}z_{i}\right\|}_{2}^{3}\;=\;O(1)\sum_{i=1}^{n}{\left(z_{i}^{T}A_{n}^{-1}z_{i}\right)}^{3/2}\;=\;o(1).$$

Thus an application of Lyapunov’s theorem yields

(25)
$${\left(u^{T}A_{n}^{-1}u\right)}^{-1/2}u^{T}A_{n}^{-1}\xi \;=\;\sum_{i=1}^{n}\eta_{i}\overset{D}{\to }N(0,\phi ).$$

By Slutsky’s lemma, we see from (23)–(25) that

$${\left(u^{T}A_{n}^{-1}u\right)}^{-1/2}\left(u^{T}\hat{\beta }-u^{T}\beta_{0}\right)\overset{D}{\to }N(0,\phi ),$$

showing the asymptotic normality of any *L*_1_-sparse one-dimensional projection $u^{T}\hat{\beta }$ of the MLE $\hat{\beta }$. This completes the proof of Theorem 1.

### B.2. Proof of Theorem 4

The proof is similar to that for Theorem 1. Without loss of generality, we assume that **Σ** = *I*_*p*_ because under global null, a rotation of **X** yields standard normal rows. First let ***ξ*** = (*ξ*_1_, ⋯ , *ξ*_*p*_)^*T*^ = (**X**^*T*^**X**)^−1^**X**^*T*^[**y** − ***μ***_0_], where ***μ***_0_ = *b′* (0)**1** with $1={\left(1,\;\cdots \;,1\right)}^{T}\;\in {\mathbb{R}}^{n}$ because ***β***_0_ = 0. Then **y** – ***μ***_0_ has i.i.d. uniform sub-Gaussian components and is independent of $X=\left(z_{1},\cdots ,z_{p}\right)\in {\mathbb{R}}^{n\times p}$. Define event

$$E\;=\;\left\{{\left\|\xi \right\|}_{\infty }\;\leq c_{2}\sqrt{n^{-1}\;\text{log}\;n}\right\}.$$

By Lemma 5, it is seen that $P(E)\;\geq \;1\;-\;o\left(p^{-a}\right)$. Furthermore, define the neighborhood

(26)
$$N_{0}\;=\;\left\{{\left\|\beta \right\|}_{\infty }\;\leq c_{3}\sqrt{n^{-1}\;\text{log}\;n}\right\}$$

for some *c*_3_ > *c*_2_(*b*″ (0))^−1^. We next show that the MLE must fall into the region $N_{0}$ with probability at least 1 − *O*(*p*^−*a*^) following the similar arguments in Theorem 1.

First, we define

$$\gamma (\beta )\;=\;{\left(\gamma_{1}(\beta ),\;\cdots \;,\gamma_{p}(\beta )\right)}^{T}\;\equiv X^{T}\mu (X\beta )$$

and

$$\Psi (\beta )\;=\;\gamma (\beta )\;-\;\gamma \left(\beta_{0}\right)\;-\;X^{T}\left[y-\mu_{0}\right],\;\beta \in {\mathbb{R}}^{p}.$$

Applying a forth order Taylor expansion of ***γ***(***β***) around ***β***_0_ = **0** with the Lagrange remainder term componentwise leads to

$$\gamma (\beta )\;=\;\gamma \left(\beta_{0}\right)+b^{\prime \prime }(0)X^{T}X\left(\beta -\beta_{0}\right)+r+s+t,$$

where **r** = (*r*_1_, ⋯ , *r*_*p*_)^*T*^, s = (*s*_1_, ⋯ , *s*_*p*_)^*T*^, **t** = (*t*_1_, ⋯ , *t*_*p*_)^*T*^ and for each 1 ≤ *j* ≤ *p*,

(27)
$$r_{j}=\frac{b^{\prime \prime \prime }(0)}{2}\sum_{i=1}^{n}x_{ij}{\left(x_{i}^{T}\beta \right)}^{2}$$

(28)
$$s_{j}=\frac{b^{\left(4\right)}(0)}{6}\sum_{i=1}^{n}x_{ij}{\left(x_{i}^{T}\beta \right)}^{3}$$

(29)
$$t_{j}=\frac{1}{24}\sum_{i=1}^{n}b^{\left(5\right)}\left(x_{i}^{T}\tilde{\beta^{j}}\right)x_{ij}{\left(x_{i}^{T}\beta \right)}^{4}.$$

With $\tilde{\beta^{j}}$ some *p*-dimensional vector lying on the line segment joining ***β*** and ***β***_0_.

Let us define another vector-valued function

(30)
$$\bar{\Psi }(\beta )\;\equiv \;{\left[b^{\prime \prime }(0)X^{T}X\right]}^{-1}\Psi (\beta )\;=\;\beta -\beta_{0}+u,$$

where **u** = −(*b*″ (0))^−1^***ξ*** + [*b*″ (0)**X**^*T*^**X**]^−1^(**r** + **s** + **t**). It follows from the above derivation that for any $\beta \in N_{0}$,

$${\left\|u\right\|}_{\infty }\;\leq \;{\left\|{\left(b^{\prime \prime }(0)\right)}^{-1}\xi \right\|}_{\infty }\;+\;{\left\|{\left[b^{\prime \prime }(0)X^{T}X\right]}^{-1}(r+s+t)\right\|}_{\infty }.$$

Now, we bound the terms on the right hand side.

First note that on event E,

(31)
$${\left\|{\left(b^{\prime \prime }(0)\right)}^{-1}\xi \right\|}_{\infty }\;\leq \;{\left(b^{\prime \prime }(0)\right)}^{-1}c_{2}\sqrt{n\;\text{log}\;n}.$$

Then, we consider the next term: ${\left\|{\left[b^{\prime \prime }(0)X^{T}X\right]}^{-1}(r+s+t)\right\|}_{\infty }$. We observe that

$${\left\|{\left[b^{\prime \prime }(0)X^{T}X\right]}^{-1}(r+s+t)\right\|}_{\infty }\;\leq {\left|b^{\prime \prime }(0)\right|}^{-1}{\left\|{\left[n^{-1}X^{T}X\right]}^{-1}\right\|}_{\infty }{\left\|n^{-1}(r+s+t)\right\|}_{\infty }\;\leq {\left|b^{\prime \prime }(0)\right|}^{-1}{\left\|{\left[n^{-1}X^{T}X\right]}^{-1}\right\|}_{\infty }\;\cdot \left({\left\|n^{-1}r\right\|}_{\infty }+{\left\|n^{-1}s\right\|}_{\infty }+{\left\|n^{-1}t\right\|}_{\infty }\right).$$

By Lemma 6, we have that ${\left\|{\left[n^{-1}X^{T}X\right]}^{-1}\right\|}_{\infty }\leq 1+O\left(pn^{-1/2}\right)$. Lemmas 10, 11, 12 assert that

$$\left({\left\|n^{-1}r\right\|}_{\infty }\;+\;{\left\|n^{-1}s\right\|}_{\infty }\;+\;{\left\|n^{-1}t\right\|}_{\infty }\right)\;=\;\left\{n^{\alpha -5/6}\;\text{log}\;n\;+\;n^{3/2\alpha -5/4}{\left(\text{log}\;n\right)}^{3/2}\;+\;n^{\alpha -1}{\left(\text{log}\;n\right)}^{1/2}\;+\;n^{2\alpha -3/2}{\left(\text{log}\;n\right)}^{3/2}\right\}\sqrt{n^{-1}\;\text{log}\;n}.$$

We combine last two bounds so that we have

(32)
$${\left\|{\left[b^{\prime \prime }(0)X^{T}X\right]}^{-1}(r+s+t)\right\|}_{\infty }\;=\;o(\sqrt{n^{-1}\;\text{log}\;n})$$

with probability at least 1 − *o*(*p*^−*c*^) when *p* = *O*(*n*^*α*^) with *α* < 2/3.

Combining equations (31) and (32), we obtain that if *p* = *O*(*n*^*α*^) with *α* ∈ [0, 2/3), then

$${\left\|u\right\|}_{\infty }\;\leq \;c_{3}\sqrt{n^{-1}\;\text{log}\;n}.$$

Thus, the MLE must fall into the region $N_{0}$ following the similar arguments in Theorem 1.

Next, we show the componentwise asymptotic normality of the MLE $\hat{\beta }$. By equation (30), we have $\hat{\beta }\;=\;-u\;=\;{\left(b^{\prime \prime }(0)\right)}^{-1}{\left(X^{T}X\right)}^{-1}X^{T}\left[y-\mu_{0}\right]\;-\;{\left[b^{\prime \prime }(0)X^{T}X\right]}^{-1}(r+s+t)$. So, we can write

(33)
$${\hat{\beta }}_{j}\;=\;{\left(b^{\prime \prime }(0)\right)}^{-1}n^{-1}e_{j}^{T}X^{T}\left[y-\mu_{0}\right]\;+\;{\left(b^{\prime \prime }(0)\right)}^{-1}T-e_{j}^{T}{\left[b^{\prime \prime }(0)X^{T}X\right]}^{-1}(r+s+t)$$

where $T\;=\;e_{j}^{T}{\left(X^{T}X\right)}^{-1}X^{T}\left[y-\mu_{0}\right]-n^{-1}e_{j}^{T}X^{T}\left[y-\mu_{0}\right]$. By Lemma 13 and Equation (32), both *n*^1/2^(*b*″ (0))^−1^*T* and $n^{1/2}e_{j}^{T}{\left[b^{\prime \prime }(0)X^{T}X\right]}^{-1}(r+s+t)$ converges to zero in probability. So, it is enough to consider the first summand in (33). Now, we show that $n^{-1/2}e_{j}^{T}X^{T}\left[y-\mu_{0}\right]$ is asymptotically normal. In fact, we can write $e_{j}^{T}X^{T}\left[y-\mu_{0}\right]=\sum_{i=1}^{n}x_{ij}y_{i}$ where each summand *x*_*ij*_*y*_*i*_ is independent over *i* and has variance *ϕb*″ (0). Moreover, $\sum_{i=1}^{n}E{\left|x_{ij}y_{i}\right|}^{3}=O(n)$ since |*x*_*ij*_|^3^ and |*y*_*i*_|^3^ are independent and finite mean. So, we apply Lyapunov’s theorem to obtain $b^{\prime \prime }{\left(0\right)}^{-1/2}n^{-1/2}e_{j}^{T}X^{T}\left[y-\mu_{0}\right]\overset{D}{\to }N\left(0,\phi \right)$. Finally, we know that $b^{\prime \prime }(0)n{\left(A_{n}^{-1}\right)}_{jj}\to 1$ in probability from the remark in Theorem 1. Thus, Slutsky’s lemma yields

(34)
$${\left(A_{n}^{-1}\right)}_{jj}^{-1/2}\;{\hat{\beta }}_{j}\overset{D}{\to }N(0,\phi ).$$

This completes the proof of the theorem.

#### Lemma 5

Assume that the components of **y** − ***μ***_0_ are uniform sub-Gaussians. That is, there exist a positive constant *C* such that *P*(|(**y** − ***μ***_0_)_*i*_| > *t*) ≤ *C* exp {−*Ct*^2^} for all 1 ≤ *i* ≤ *n*. Then, it holds that, for some positive constant *c*_2_,

$${\left\|{\left(X^{T}X\right)}^{-1}X^{T}\left(y-\mu_{0}\right)\right\|}_{\infty }\;\leq \;c_{2}\sqrt{n^{-1}\;\text{log}\;n}.$$

with asymptotic probability 1 − *o*(*p*^−*a*^).

**Proof** We prove the result by conditioning on **X**. Let **E** = *n*^−1^**X**^*T*^**X** − **I**_*p*_. Then by matrix inversion,

$${\left(n^{-1}X^{T}X\right)}^{-1}\;=\;{\left(I_{p}\;+\;E\right)}^{-1}\;=\;I_{p}\;-\;\sum_{k=1}^{\infty }{\left(-1\right)}^{k+1}{\left(E\right)}^{k}\;=\;I_{p}\;-\;E\;+\;\sum_{k=2}^{\infty }{\left(-1\right)}^{k}{\left(E\right)}^{k}\;=\;2I_{p}\;-\;n^{-1}X^{T}X\;+\;\sum_{k=2}^{\infty }{\left(-1\right)}^{k}{\left(E\right)}^{k}.$$

Thus, it follows that

$${\left\|{\left(X^{T}X\right)}^{-1}X^{T}\left(y-\mu_{0}\right)\right\|}_{\infty }\;\leq \;{\left\|2n^{-1}X^{T}\left(y-\mu_{0}\right)\right\|}_{\infty }+{\left\|n^{-2}X^{T}XX^{T}\left(y-\mu_{0}\right)\right\|}_{\infty }+{\left\|n^{-1}\sum_{k=2}^{\infty }{\left(-1\right)}^{k}{\left(E\right)}^{k}X^{T}\left(y-\mu_{0}\right)\right\|}_{\infty }\;=\eta_{1}+\eta_{2}+\eta_{3}.$$

In the rest of the proof, we will bound *η*_1_, *η*_2_ and *η*_3_.

**Part 1: Bound of** *η*_1_.

First, it is easy to see that

$$\eta_{1}\;=\;{\left\|2n^{-1}X^{T}\left(y-\mu_{0}\right)\right\|}_{\infty }\;=\;2\;\underset{1\leq j\leq p}{\text{max}}\left|n^{-1}\sum_{i=1}^{n}x_{ij}{\left(y-\mu_{0}\right)}_{i}\right|.$$

We observe that each summand *x*_*ij*_(**y** − ***μ***_0_)_*i*_ is the product of two subgaussian random variables, and so satisfies *P*(|*x*_*ij*_(**y** *−* ***μ***_0_)_*i*_| > *t*) ≤ *C* exp(−*Ct*) for some constant *C* > 0 by Lemma 1 in Fan et al. (2016). Moreover, *E*[*x*_*ij*_(**y** − ***μ***_0_)_*i*_] = 0 since *x*_*ij*_ and (**y** − ***μ***_0_)_*i*_ are independent and have zero mean. Thus, we can use Lemma 9 by setting *W*_*ij*_ = *x*_*ij*_(**y** − ***μ***_0_)_*i*_ and *α* = 1. So, we get

(35)
$$\eta_{1}\;=\;2\underset{1\leq j\leq p}{\text{max}}\left|n^{-1}\sum_{i=1}^{n}x_{ij}{\left(y-\mu_{0}\right)}_{i}\right|\;\leq \;c_{2}\sqrt{n^{-1}\;\text{log}\;p}$$

with probability 1 − *O*(*p*^−*c*^) for some positive constants *c* and *c*_2_.

**Part 2: Bound of** *η*_2_.

Now, we study *η*_2_ = ‖*n*^−2^**X**^*T*^**XX**^*T*^(**y** − ***μ***_0_)‖_∞_. Let **z**_*k*_ be the *k*-th column of *X*, that is **z**_*k*_ = **X**e_*k*_. Direct calculations yield

$$e_{k}^{T}X^{T}XX^{T}\left(y-\mu_{0}\right)\;=\;\sum_{j=1}^{p}\left(z_{k}^{T}z_{j}\right)\left(z_{j}^{T}\left(y-\mu_{0}\right)\right)\;=\;{\left\|z_{k}\right\|}_{2}^{2}z_{k}^{T}\left(y-\mu_{0}\right)\;+\;\sum_{j\neq k}^{p}\left(z_{k}^{T}z_{j}\right)\left(z_{j}^{T}\left(y-\mu_{0}\right)\right).$$

Thus, it follows that

(36)
$${\left\|X^{T}XX^{T}\left(y-\mu_{0}\right)\right\|}_{\infty }\;\leq \;{\text{max}}_{k}\left|{\left\|z_{k}\right\|}_{2}^{2}z_{k}^{T}\left(y-\mu_{0}\right)\right|\;+\;\underset{k}{\text{max}}\left|\sum_{j\neq k}^{p}\left(z_{k}^{T}z_{j}\right)\left(z_{j}^{T}\left(y-\mu_{0}\right)\right)\right|.$$

First, we consier ${\text{max}}_{k}\left|{\left\|z_{k}\right\|}_{2}^{2}z_{k}^{T}\left(y-\mu_{0}\right)\right|$. Lemma 14 shows that ${\text{max}}_{k}{\left\|z_{k}\right\|}_{2}^{2}\leq O(n)$ with probability 1 − *O*(*p*^−*c*^). We also have ${\text{max}}_{k}\left|z_{k}^{T}\left(y-\mu_{0}\right)\right|=\frac{n}{2}\eta_{1}\leq O\left(\sqrt{n\;\text{log}\;p}\right)$ by equation (35). It follows that

(37)
$$\underset{k}{\text{max}}\left|\|z_{k}{\|}_{2}^{2}z_{k}^{T}\left(y-\mu_{0}\right)\right|\;\leq \;{\text{max}}_{k}\|z_{k}{\|}_{2}^{2}{\text{max}}_{k}\left|z_{k}^{T}\left(y-\mu_{0}\right)\right|\;\leq \;O(n\sqrt{n\;\text{log}\;p}).$$

Next, let $a_{j}\;=\;z_{k}^{T}z_{j}/{\left\|z_{k}\right\|}_{2}$ and $b_{j}\;=\;z_{j}^{T}\left(y-\mu_{0}\right)/{\left\|y-\mu_{0}\right\|}_{2}$. Then it is easy to see that conditional on **z**_*k*_ and **y**, *a*_*j*_ ~ *N*(0, 1), *b*_*j*_ ~ *N*(0, 1) and $\mathrm{cov}\left(a_{j},b_{j}|z_{k},y\right)\;=\;z_{k}^{T}\left(y-\mu_{0}\right)/\left({\left\|z_{k}\right\|}_{2}\;{\left\|y\;-\;\mu_{0}\right\|}_{2}\right)$. By (E.6) of Lemma 7 in Fan et al. (2016), it can be shown that

$$P\left(\frac{1}{p-1}\left|\sum_{j\neq k}^{p}\left(z_{k}^{T}z_{j}\right)\left(z_{j}^{T}\left(y-\mu_{0}\right)\right)-z_{k}^{T}\left(y-\mu_{0}\right)\right|\geq c{\left\|z_{k}\right\|}_{2}{\left\|y-\mu_{0}\right\|}_{2}\sqrt{p^{-1}\;\text{log}\;p}|z_{k},y\right)\;=P\left(\frac{1}{p-1}\left|\sum_{j\neq k}^{p}a_{j}b_{j}\;-\;\frac{z_{k}^{T}\left(y-\mu_{0}\right)}{{\left\|z_{k}\right\|}_{2}{\left\|y-\mu_{0}\right\|}_{2}}\right|\;\geq \;c\sqrt{p^{-1}\;\text{log}\;p}|z_{k},y\right)\;\leq \;cp^{-c_{1}},$$

where *c*_1_ is some large positive constant independent of **z**_*k*_ and **y**. Moreover, we can choose *c*_1_ as large as we want by increasing *c*. Thus, it follows that

$$P\left(\frac{1}{p-1}\left|\sum_{j\neq k}^{p}\left(z_{k}^{T}z_{j}\right)\left(z_{j}^{T}\left(y-\mu_{0}\right)\right)-z_{k}^{T}\left(y-\mu_{0}\right)\right|\geq c{\left\|z_{k}\right\|}_{2}{\left\|y-\mu_{0}\right\|}_{2}\sqrt{p^{-1}\;\text{log}\;p}\right)\leq cp^{-c_{1}}.$$

It follows from probability union bound that

$$P\left(\frac{1}{p-1}{\text{max}}_{k}\left|\frac{1}{{\left\|z_{k}\right\|}_{2}{\left\|y-\mu_{0}\right\|}_{2}}\sum_{j\neq k}^{p}\left(z_{k}^{T}z_{j}\right)\left(z_{j}^{T}\left(y-\mu_{0}\right)\right)-z_{k}^{T}\left(y-\mu_{0}\right)\right|\;\geq \;c\sqrt{p^{-1}\text{log}\;p}\right)\leq cp^{-c_{1}+1}.$$

Taking *c*_1_ > 1 yields that with probability at least 1 − *o*(*p*^−*a*^) for some *a* > 0,

$$\underset{k}{\text{max}}\left\{\frac{1}{{\left\|z_{k}\right\|}_{2}{\left\|y-\mu_{0}\right\|}_{2}}\left|\frac{1}{p-1}\sum_{j\neq k}^{p}\left(z_{k}^{T}z_{j}\right)\left(z_{j}^{T}\left(y-\mu_{0}\right)\right)-z_{k}^{T}\left(y-\mu_{0}\right)\right|\right\}\;\leq \;c\sqrt{p^{-1}\;\text{log}\;p}.$$

By Lemma 14, we have ${\text{max}}_{k}\|z_{k}{\|}_{2}\;=\;\sqrt{{\text{max}}_{k}\|z_{k}{\|}_{2}^{2}}\leq O_{p}\left(\sqrt{n}\right)$. Therefore, by using the fact that ${\left\|y-\mu_{0}\right\|}_{2}\leq O_{p}\left(\sqrt{n}\right)$, we have

(38)
$$\underset{k}{\text{max}}\left|\sum_{j\neq k}^{p}\left(z_{k}^{T}z_{j}\right)\left(z_{j}^{T}\left(y-\mu_{0}\right)\right)\right|\;\leq \underset{k}{\text{max}}\left|\sum_{j\neq k}^{p}\left[\left(z_{k}^{T}z_{j}\right)\left(z_{j}^{T}\left(y-\mu_{0}\right)\right)\right]-(p-1)z_{k}^{T}\left(y-\mu_{0}\right)\right|+(p-1)\underset{k}{\text{max}}\left|z_{k}^{T}\left(y-\mu_{0}\right)\right|\;\leq p\;\underset{k}{\text{max}}{\left\|z_{k}\right\|}_{2}{\left\|y-\mu_{0}\right\|}_{2}\underset{k}{\text{max}}\left\{\frac{1}{{\left\|z_{k}\right\|}_{2}{\left\|y-\mu_{0}\right\|}_{2}}|\frac{1}{p-1}\sum_{j\neq k}^{p}\left[\left(z_{k}^{T}z_{j}\right)\left(z_{j}^{T}\left(y-\mu_{0}\right)\right)-z_{k}^{T}\left(y-\mu_{0}\right)\right]\right\}+p\;\underset{k}{\text{max}}\left|z_{k}^{T}\left(y-\mu_{0}\right)\right|\;\leq cpn\sqrt{\text{log}\;p}\sqrt{p^{-1}\;\text{log}\;p}+cp\sqrt{n\;\text{log}\;p}.$$

Combining (36)–(38) yields

(39)
$$\eta_{2}\;=\;{\left\|n^{-2}X^{T}XX^{T}\left(y-\mu_{0}\right)\right\|}_{\infty }\;\leq \;cp^{1/2}n^{-1}\;\text{log}\;p\;=\;o(\sqrt{n^{-1}\;\text{log}\;n}).$$

**Part 3: Bound of** *η*_3_.

Finally, we study *η*_3_. We observe that $\eta_{3}\leq \|\sum_{k=2}^{\infty }{\left(-1\right)}^{k\;+\;1}{\left(E\right)}^{k}{\|}_{\infty }\|n^{-1}X^{T}\left(y-\mu_{0}\right){\|}_{\infty }$. Lemma 7 proves that $\|\sum_{k=2}^{\infty }{\left(-1\right)}^{k\;+\;1}{\left(E\right)}^{k}{\|}_{\infty }\leq O\left(p^{3/2}n^{-1}\right)$ while equation (35) shows that ${\left\|n^{-1}X^{T}\left(y-\mu_{0}\right)\right\|}_{\infty }\;=\;O\left(\sqrt{n^{-1}\;\text{log}\;p}\right)$ with probability 1 − *O*(*p*^−*c*^). Putting these facts together, we obtain

(40)
$$\eta_{3}\leq O\left(p^{3/2}n^{-1}\sqrt{n^{-1}\;\text{log}\;p}\right)\;=\;o(\sqrt{n^{-1}\;\text{log}\;n})$$

where we use $p=O\left(n^{\alpha_{0}}\right)$ with *α*_0_ ∈ [0, 2/3).

Combining equations (35), (39), and (40), we obtain that with probability at least 1 − *o*(*p*^−*a*^),

$${\left\|{\left(X^{T}X\right)}^{-1}X^{T}\left(y-\mu_{0}\right)\right\|}_{\infty }\;\leq \;c\sqrt{n^{-1}\;\text{log}\;n}.$$

■

#### Lemma 6

Under the assumptions of Theorem 4, ‖(*n*^−1^**X**^*T*^**X**)^−1^‖_∞_ ≤ 1 + *O*(*pn*^−1/2^) with probability 1 − *O*(*p*^−*c*^).

**Proof** Let **E** = *n*^−1^**X**^*T*^**X** − **I**_*p*_. Then, ‖**E**‖_2_ ≤ *C*(*p*/*n*)^1/2^ for some constant *C* with probability 1 − *O*(*p*^−*c*^) by Theorem 4.6.1 in Vershynin (2016). Furthermore, by matrix inversion, we get

$${\left(n^{-1}X^{T}X\right)}^{-1}\;=\;{\left(I_{p}+E\right)}^{-1}\;=\;I_{p}\;-\;\sum_{k=1}^{\infty }{\left(-1\right)}^{k+1}{\left(E\right)}^{k}.$$

Now, we take the norm and use triangle inequalities to get

$${\left\|{\left(n^{-1}X^{T}X\right)}^{-1}\right\|}_{\infty }\;\leq \;{\left\|I_{p}\right\|}_{\infty }\;+\;\sum_{k=1}^{\infty }{\left\|E^{k}\right\|}_{\infty }\;\leq \;1\;+\;p^{1/2}\sum_{k=1}^{\infty }{\left\|E^{k}\right\|}_{2}\;\leq \;1\;+\;p^{1/2}\sum_{k=1}^{\infty }\|E{\|}_{2}^{k}\;\leq \;1\;+\;Cp^{1/2}\sum_{k=1}^{\infty }{\left({\left(p/n\right)}^{1/2}\right)}^{k}\;\leq \;1\;+\;Cp^{1/2}{\left(p/n\right)}^{1/2}$$

where we use the fact that *p*/*n* is bounded by a constant less than 1. ■

#### Lemma 7

In the same setting as Lemma 6, if **E** = *n*^−1^**X**^*T*^**X**−**I**_*p*_ then ${\left\|\sum_{k=2}^{\infty }{\left(-1\right)}^{k+1}{\left(E\right)}^{k}\right\|}_{\infty }\;\leq \;Cp^{3/2}n^{-1}$, with probability 1 − *O*(*p*^−*c*^).

**Proof** Again, we use that ‖**E**‖_2_ ≤ *C*(*p*/*n*)^1/2^ for some constant *C* with probability 1 − *O*(*p*^−*c*^). By similar calculations as in Lemma 6, we deduce

$${\left\|\sum_{k=2}^{\infty }{\left(-1\right)}^{k+1}{\left(E\right)}^{k}\right\|}_{\infty }\leq \sum_{k=2}^{\infty }{\left\|{\left(-1\right)}^{k+1}{\left(E\right)}^{k}\right\|}_{\infty }\;\leq \;\sum_{k=2}^{\infty }p^{1/2}{\left\|{\left(E\right)}^{k}\right\|}_{2}\;=\;\sum_{k=2}^{\infty }p^{1/2}\|E{\|}_{2}^{k}\;\leq \sum_{k=2}^{\infty }p^{1/2}{\left(p/n\right)}^{k/2}\;\leq \;Cp^{3/2}n^{-1}.$$

■

#### Lemma 8

Let **W**_*j*_ be nonnegative random variables for 1 ≤ *j* ≤ *p* that are not necessarily independent. If *P* (*W*_*j*_ > *t*) ≤ *C*_1_ exp(−*C*_2_*a*_*n*_*t*^2^) for some constants *C*_1_ and *C*_2_ and for some sequence *a*_*n*_, then for any *c* > 0, ${\text{max}}_{1\leq j\leq p}\;W_{j}\;\leq \;{\left((c+1)/C_{2}\right)}^{1/2}\;a_{n}^{-1/2}{\left(\text{log}\;p\right)}^{1/2}$ with probability at least 1 − *O*(*p*^−*c*^).

**Proof** Using union bound, we get

$$P\left(\underset{1\leq j\leq p}{\text{max}}W_{j}>t\right)\leq \sum_{1\leq j\leq p}P\left(W_{j}>t\right)\leq pC_{1}\text{exp}\left(-C_{2}a_{n}t^{2}\right).$$

Taking $t=a_{n}^{-1/2}{\left(\text{log}\;p\right)}^{1/2}{\left((c+1)/C_{2}\right)}^{1/2}$ concludes the proof since then

$$P\left(\underset{1\leq j\leq p}{\text{max}}\;W_{j}>a_{n}^{-1/2}{\left(\text{log}\;p\right)}^{1/2}{\left((c+1)/C_{2}\right)}^{1/2}\right)\leq C_{1}p^{-c}.$$

■

#### Lemma 9

Let *W*_*ij*_ be random variables which are independent over the index *i*. Assume that there are constants *C*_1_ and *C*_2_ such that $P\left(\left|W_{ij}\right|>t\right)\;\leq \;C_{1}\text{exp}\left(-C_{2}t^{\alpha }\right)$ with 0 < *α* ≤ 1. Then, with probability 1 − *O*(*p*^−c^),

$$\underset{0\leq j\leq p}{\text{max}}\left|n^{-1}\overset{n}{\underset{i=1}{\sum }}\left(W_{ij}-EW_{ij}\right)\right|\;\leq \;Cn^{-(1/2)\alpha }{\left(\text{log}\;p\right)}^{1/2},$$

for some positive constants *c* and *C*.

**Proof** We have $P\left(\left|n^{-1}\sum_{i=1}^{n}\left(W_{ij}-EW_{ij}\right)\right|\;>\;t\right)\;\leq \;C_{3}\text{exp}\left(-C_{4}n^{\alpha }t^{2}\right)$ by Lemma 6 of Fan et al. (2016) where *C*_3_ and *C*_4_ are some positive constants which only depend on *C*_1_ and *C*_2_. This probability bound shows that the assumption of Lemma 8 holds with *a*_*n*_ = *n*^*α*^. Thus, Lemma 8 finishes the proof. ■

#### Lemma 10

With probability 1 − *O*(*p*^−*c*^), the vector **r** defined in (27) satisfies the bound ${\left\|n^{-1}r\right\|}_{\infty }\;=\;O\left(n^{\alpha -5/6}\text{log}\;n\sqrt{n^{-1}\text{log}\;n}\right)$.

**Proof** We begin by observing that both *x*_*ij*_ and $\left(x_{i}^{T}\beta /\|\beta {\|}_{2}\right)$ are standard normal variables. So, using Lemma 1 of Fan et al. (2016), we have $P\left(x_{ij}{\left(x_{i}^{T}\beta /\|\beta {\|}_{2}\right)}^{2}>t\right)\;\leq \;C\;\text{exp}\left(-Ct^{2/3}\right)$ for some constant *C* which does not depend ***β***. It is easy to see that $x_{ij}{\left(x_{i}^{T}\beta \right)}^{2}$ are independent random variables across *i*’s with mean 0. By Lemma 9, ${\text{max}}_{1\leq j\leq p}\left|n^{-1}\sum_{i=1}^{n}x_{ij}{\left(\frac{x_{i}^{T}\beta }{\|\beta {\|}_{2}}\right)}^{2}\right|$ is of order *O*(*n*^−1/3^(log *p*)^1/2^). Moreover, $\|\beta {\|}_{2}\leq p^{1/2}\|\beta {\|}_{\infty }\;\leq \;O\left(p^{1/2}\sqrt{n^{-1}\;\text{log}\;n}\right)$ when $\beta \in N_{0}$. Therefore,

$${\left\|n^{-1}r\right\|}_{\infty }\;=\;\underset{1\leq j\leq p}{\text{max}}\left|\frac{b^{\left(3\right)}(0)}{2}\|\beta {\|}_{2}^{2}n^{-1}\sum_{i=1}^{n}x_{ij}{\left(x_{i}^{T}\beta /\|\beta {\|}_{2}\right)}^{2}\right|\;\leq Cpn^{-1}\left(\text{log}\;n\right)n^{-1/3}{\left(\text{log}\;p\right)}^{1/2}=Cn^{\alpha -4/3}{\left(\text{log}\;n\right)}^{3/2}\;=O\left(n^{\alpha -5/6}\left(\text{log}\;n\right)\sqrt{n^{-1}\text{log}\;n}\right),$$

since *p* = *O*(*n*^*α*^). ■

#### Lemma 11

With probability 1 − *O*(*p*^−*c*^), the vector **s** defined in (28) satisfies the bound ${\left\|n^{-1}s\right\|}_{\infty }\;=\;O\left(\left(n^{3/2\alpha -5/4}{\left(\text{log}\;n\right)}^{3/2}\right)\;+\;\left(n^{\alpha -1}{\left(\text{log}\;n\right)}^{1/2}\right)\sqrt{n^{-1}\;\text{log}\;n}\right)$.

**Proof** First, observe that for some constant *C*, $\left|n^{-1}s_{j}\right|\leq C\|\beta {\|}_{2}^{3}n^{-1}\sum_{i=1}^{n}x_{ij}{\left(\frac{x_{i}^{T}\beta }{\|\beta {\|}_{2}}\right)}^{3}$. Moreover, the summands $x_{ij}{\left(\frac{x_{i}^{T}\beta }{\|\beta {\|}_{2}}\right)}^{3}$ are independent over *i* and they satisfy the probability bound $P\left(\left|x_{ij}{\left(\frac{x_{i}^{T}\beta }{\|\beta {\|}_{2}}\right)}^{3}\right|>t\right)\leq C\text{exp}\left(-Ct^{1/2}\right)$ by Lemma 1 of Fan et al. (2016). Thus, by Lemma 9, we obtain

$$\underset{1\leq j\leq p}{\text{max}}\left|n^{-1}\sum_{i=1}^{n}\left(x_{ij}{\left(\frac{x_{i}^{T}\beta }{\|\beta {\|}_{2}}\right)}^{3}\;-\;E\left[{\left(x_{ij}\frac{x_{i}^{T}\beta }{\|\beta {\|}_{2}}\right)}^{3}\right]\right)\right|\;=\;O\left(n^{-1/4}{\left(\text{log}\;p\right)}^{1/2}\right).$$

Now, we calculate the expected value of the summand $x_{ij}{\left(\frac{X_{i}^{T}\beta }{\|\beta {\|}_{2}}\right)}^{3}$. We decompose $X_{i}^{T}\beta$ as $x_{ij}\beta_{j}\;+\;x_{i,-j}^{T}\beta_{-j}$ where **x**_*i*,−*j*_ and ***β***_−*j*_ are the vectors **x**_*i*_ and ***β*** whose *j*th entry is removed. We use the independence of **x**_*i*,−*j*_ and *x*_*ij*_ and get

$$E\left[x_{ij}{\left(\frac{x_{i}^{T}\beta }{\|\beta {\|}_{2}}\right)}^{3}\right]\;=\;\frac{1}{\|\beta {\|}_{2}^{3}}E\left[x_{ij}{\left(x_{ij}\beta_{j}+x_{i,-j}^{T}\beta_{-j}\right)}^{3}\right]\;=\;\frac{1}{\|\beta {\|}_{2}^{3}}E\left[x_{ij}^{4}\beta_{j}^{3}\;+\;3x_{ij}^{3}\beta_{j}^{2}\left(x_{i,-j}^{T}\beta_{-j}\right)\;+\;3x_{ij}^{2}\beta_{j}{\left(x_{i,-j}^{T}\beta_{-j}\right)}^{2}\;+\;x_{ij}{\left(x_{i,-j}^{T}\beta_{-j}\right)}^{3}\right]\;=\;\frac{1}{\|\beta {\|}_{2}^{3}}\left[3\beta_{j}^{3}\;+\;3\beta_{j}{\left\|\beta_{-j}\right\|}_{2}^{2}\right]\;=\frac{3\beta_{j}}{\|\beta {\|}_{2}}.$$

Finally, we can combine the result of Lemma 9 and the expected value of $x_{ij}{\left(\frac{x_{i}^{T}\beta }{\|\beta {\|}_{2}}\right)}^{3}$. we bound ‖*n*^−1^**s**‖_∞_ as follows

$${\left\|n^{-1}s\right\|}_{\infty }\leq C\|\beta {\|}_{2}^{3}\underset{1\leq j\leq p}{\text{max}}\left|n^{-1}\sum_{i=1}^{n}x_{ij}{\left(\frac{{\text{x}}_{i}^{T}\beta }{\|\beta {\|}_{2}}\right)}^{3}\right|\;\leq \;O\left(\|\beta {\|}_{2}^{3}\left(n^{-1/4}{\left(\text{log}\;p\right)}^{1/2}+\frac{\|\beta {\|}_{\infty }}{\|\beta {\|}_{2}}\right)\right)\;\leq \;O\left(\|\beta {\|}_{2}^{3}n^{-1/4}{\left(\text{log}\;p\right)}^{1/2}+\|\beta {\|}_{\infty }\|\beta {\|}_{2}^{2}\right)).$$

Since $\beta \in N_{0}$, we have ‖***β***‖_2_ = *O*(*p*^1/2^*n*^−1/2^(log *p*)^1/2^) and ‖***β***‖_∞_ = *O*(*n*^−1/2^(log *p*)^1/2^). Thus, ${\left\|n^{-1}s\right\|}_{\infty }=O(\left(n^{3/2\alpha -5/4}{\left(\text{log}\;n\right)}^{3/2}+\left(n^{\alpha -1}{\left(\text{log}\;n\right)}^{1/2}\right)\sqrt{n^{-1}\text{log}\;n}\right)$ when *p* = *O*(*n*^*α*^). ■

#### Lemma 12

With probability 1 − *O*(*p*^−*c*^), the vector **t** defined in (29) satisfies the bound ${\left\|n^{-1}t\right\|}_{\infty }=O\left(n^{2\alpha -3/2}{\left(\text{log}\;n\right)}^{3/2}\sqrt{n^{-1}\text{log}\;n}\right)$.

**Proof** The proof is similar to the proof of Lemma 11. Since *b*^(5)^(·) is uniformly bounded, $\left|n^{-1}t_{j}\right|\;\leq \;C\|\beta {\|}_{2}^{4}\;n^{-1}\sum_{i=1}^{n}\left|x_{ij}{\left(\frac{x_{i}^{T}\beta }{\|\beta {\|}_{2}}\right)}^{4}\right|$ for some constant C. We focus on the summands $\left|x_{ij}{\left(\frac{x_{i}^{T}\beta }{\|\beta {\|}_{2}}\right)}^{4}\right|$ which are independent across *i*. Moreover, repeated application of Lemma 1 of Fan et al. (2016) yields $P\left(x_{ij}{\left(x_{i}^{T}\beta /\|\beta {\|}_{2}\right)}^{2}\;>\;t\right)\;\leq \;C\text{exp}\left(-Ct^{2/5}\right)$ for some constant *C* independent of ***β***. We can bound the expected value of the summand by Cauchy-Schwartz: $E\left[\left|x_{ij}{\left(\frac{x_{i}^{T}\beta }{\|\beta {\|}_{2}}\right)}^{4}\right|\right]\;\leq \;{\left(Ex_{ij}^{2}E\left[{\left(\frac{x_{i}^{T}\beta }{\|\beta {\|}_{2}}\right)}^{8}\right]\right)}^{1/2}\;=\;\sqrt{105}$. So, by Lemma 9, we get

$${\left\|n^{-1}t\right\|}_{\infty }\leq C\|\beta {\|}_{2}^{4}\left(\sqrt{105}+n^{-1/5}{\left(\text{log}\;p\right)}^{1/2}\right)\;=O\left(\|\beta {\|}_{2}^{4}\right)=O\left(p^{2}n^{-2}{\left(\text{log}\;n\right)}^{2}\right).$$

Finally, we can deduce that ${\left\|n^{-1}t\right\|}_{\infty }\;=\;O\left(n^{2\alpha -3/2}{\left(\text{log}\;n\right)}^{3/2}\sqrt{n^{-1}\text{log}\;n}\right)$ when *p* = *O*(*n*^*α*^). ■

#### Lemma 13

Let $T=e_{j}^{T}{\left(X^{T}X\right)}^{-1}X^{T}\left[y-\mu_{0}\right]-n^{-1}e_{j}^{T}X^{T}\left[y-\mu_{0}\right]$. Under the assumptions of Theorem 4, we have

(41)
$$e_{j}^{T}{\left(X^{T}X\right)}^{-1}X^{T}\left[y-\mu_{0}\right]\;-\;n^{-1}e_{j}^{T}X^{T}\left[y-\mu_{0}\right]\;=\;o_{p}\left(n^{-1/2}\right).$$

**Proof** Since **X** and **y** are independent, expectation of *T* is clearly zero. Then, we consider the variance of *T*. To this end, we condition on *X*. We can calculate the conditional variance of *T* as follows

$$\text{var}[T|X]\;=\;\text{var}\left[e_{j}^{T}\left({\left(X^{T}X\right)}^{-1}-n^{-1}I_{p}\right)X^{T}\left(y-\mu_{0}\right)|X\right]\;=\phi b^{\prime \prime }(0)e_{j}^{T}\left({\left(X^{T}X\right)}^{-1}-n^{-1}I_{p}\right)X^{T}X\left({\left(X^{T}X\right)}^{-1}-n^{-1}I_{p}\right)e_{j}$$

where we use var[*y*] = *ϕb*″ (0)*I*_*n*_. When we define **E** = *n*^−1^**X**^*T*^**X** − *I*_*p*_, simple calculations show that

$$\text{Var}[T|X]\;=\;\phi b^{\prime \prime }(0)n^{-1}e_{j}^{T}\left({\left(n^{-1}X^{T}X\right)}^{-1}-I_{p}\right)+\left(n^{-1}X^{T}X-I_{p}\right))e_{j}\;=\phi b^{\prime \prime }(0)n^{-1}e_{j}^{T}\left(\sum_{k=2}^{\infty }{\left(-1\right)}^{k}E^{k}\right)e_{j}.$$

Now, we can obtain the unconditional variance using the law of total variance.

$$\text{var}[T]\;=\;E[\text{var}[T|X]]\;+\;\text{var}[E[T|X]]\;=\;\phi b^{\prime \prime }(0)n^{-1}e_{j}^{T}E\left(\sum_{k=2}^{\infty }{\left(-1\right)}^{k}E^{k}\right)e_{j}.$$

Thus, using Lemma 7, we can show that var[*T*] = *o*(*n*^−1^). Finally, we use Chebyshev’s inequality *P*(|*T*| > *n*^−1/2^) ≤ *n*var[*T*] = *o*(1). So, we conclude that *T* = *o* (*n*^−1/2^) ■

#### Lemma 14

Let *x*_*ij*_ be standard normal random variables for 1 ≤ *i* ≤ *n* and 1 ≤ *j* ≤ *p*. Then, ${\text{max}}_{1\leq j\leq p}\sum_{i=1}^{n}x_{ij}^{2}\;\leq \;n+O\left(n^{1/2}{\left(\text{log}p\right)}^{1/2}\right)$ with probability 1 − *O*(*p*^−*c*^) for some positive constant *c*. Consequently, when log *p = O(n*^*α*^) for some 0 < *α* ≤ 1, we have ${\text{max}}_{1\leq j\leq p}\sum_{i=1}^{n}x_{ij}^{2}\;=\;O(n)$, for large enough n with probability 1 – *O*(*p*^−*c*^).

**Proof** Since *x*_*ij*_ is a standard normal variable, $x_{ij}^{2}$ is subexponential random variable whose mean is 1. So, Lemma 9 entails that

$$\underset{1\leq j\leq p}{\text{max}}\left|n^{-1}\sum_{i=1}^{n}\left(x_{ij}^{2}-1\right)\right|\;=\;O\left(n^{-1/2}{\left(\text{log}p\right)}^{1/2}\right)$$

with probability 1 − *O*(*p*^−*c*^). Thus, simple calculations yields

$$\underset{1\leq j\leq p}{\text{max}}\sum_{i=1}^{n}x_{ij}^{2}\;=\;\underset{1\leq j\leq p}{\text{max}}\left|n\;+\;\sum_{i=1}^{n}\left(x_{ij}^{2}-1\right)\right|\;\leq \;n+O\left(n^{1/2}{\left(\text{log}p\right)}^{1/2}\right)$$

with probability 1 − *O*(*p*^−*c*^). ■

### B.3. Proof of Theorem 2

To prove the conclusion in Theorem 2, we use the proof by contradiction. Let us make an assumption (A) that the asymptotic normality (11) in Theorem 1 which has been proved to hold when *p* = *o*(*n*^1/2^) continues to hold when *p* ~ *n*^*α*0^ for some constant 1/2 < *α*_0_ ≤ 1, where ~ stands for asymptotic order. As shown in Section 3.1, in the case of logistic regression under global null (that is, ***β***_0_ = **0**) with deterministic rescaled orthonormal design matrix **X** (in the sense of *n*^−1^**X**^*T*^**X** = *I*_*p*_) the limiting distribution in (11) by assumption (A) becomes

(42)
$$2^{-1}n^{1/2}{\hat{\beta }}_{j}\overset{D}{\to }N(0,1),$$

where $\hat{\beta }\;=\;{\left({\hat{\beta }}_{1},\;\cdots \;,{\hat{\beta }}_{p}\right)}^{T}$ is the MLE.

Let us now assume that the rescaled random design matrix *n*^−1/2^**X** is uniformly distributed on the Stiefel manifold $V_{p}\left({\mathbb{R}}^{n}\right)$ which can be thought of as the space of all *n* × *p* orthonormal matrices. Then it follows from (42) that

(43)
$$2^{-1}n^{1/2}{\hat{\beta }}_{j}\overset{D}{\to }N(0,1)\;\text{conditional}\;\text{on}\;X.$$

Based on the limiting distribution in (43), we can make two observations. First, it holds that

(44)
$$2^{-1}n^{1/2}{\hat{\beta }}_{j}\overset{D}{\to }N\left(0,1\right)$$

unconditional on the design matrix **X**. Second, ${\hat{\beta }}_{j}$ is asymptotically independent of the design matrix **X**, and so is the MLE $\hat{\beta }$.

Since the distribution of *n*^−1/2^**X** is assumed to be the Haar measure on the Stiefel manifold $V_{p}\left({\mathbb{R}}^{n}\right)$, we have

(45)
$$n^{-1/2}XQ\;\overset{d}{=}\;n^{-1/2}X,$$

where **Q** is any fixed *p* × *p* orthogonal matrix and $\overset{d}{=}$ stands for equal in distribution. Recall that the MLE $\hat{\beta }$ solves the score equation (4), which is in turn equivalent to equation

(46)
$$Q^{T}X^{T}[y-\mu (X\beta )]=0$$

since **Q** is orthogonal. We now use the fact that the model is under global null which entails that the response vector **y** is independent of the design matrix **X**. Combining this fact with (45)–(46) yields

(47)
$$Q^{T}\hat{\beta }\overset{d}{=}\hat{\beta }$$

by noting that **X*β*** = (**XQ**)(**Q**^*T*^ ***β***). Since the distributional identity (47) holds for any fixed *p* × *p* orthogonal matrix **Q**, we conclude that the MLE $\hat{\beta }$ has a spherical distribution on ${\mathbb{R}}^{p}$. It is a well-known fact that all the marginal characteristic functions of a spherical distribution have the same generator. Such a fact along with (44) entails that

(48)
$$2^{-1}n^{1/2}\hat{\beta }\;\text{is}\;\text{asymptotically}\;\text{close}\;\text{to}\;N\left(0,I_{p}\right).$$

To simplify the exposition, let us now make the asymptotic limit exact and assume that

(49)
$$\hat{\beta }\sim N\left(0,4n^{-1}I_{p}\right)\;\text{and}\;\text{is}\;\text{independent}\;\text{of}\;X.$$

The remaining analysis focuses on the score equation (4) which is solved exactly by the MLE $\hat{\beta }$, that is,

(50)
$$X^{T}[y-\mu (X\hat{\beta })]\;=\;0,$$

which leads to

(51)
$$\xi \;\equiv \;n^{-1/2}X^{T}[y\;-\;\mu (0)]\;=\;n^{-1/2}X^{T}[\mu (X\hat{\beta })\;-\;\mu (0)]\;\equiv \;\eta .$$

Let us first consider the random variable ***ξ*** defined in (51). Note that 2[**y** − ***μ***(**0**)] has independent and identically distributed (i.i.d.) components each taking value 1 or −1 with equal probability 1/2, and is independent of **X**. Thus since *n*^−1/2^**X** is uniformly distributed on the Stiefel manifold $V_{p}\left({\mathbb{R}}^{n}\right)$, it is easy to see that

(52)
$$\xi \;=\;n^{-1/2}X^{T}[y-\mu (0)]\overset{d}{\;=}\;2^{-1}n^{-1/2}X^{T}1,$$

where $1\in {\mathbb{R}}^{n}$ is a vector with all components being one. Using similar arguments as before, we can show that ***ξ*** has a spherical distribution on ${\mathbb{R}}^{p}$. Thus the joint distribution of ***ξ*** is determined completely by the marginal distribution of ***ξ***. For each 1 ≤ *j* ≤ *p*, denote by ***ξ***_*j*_ the *j*th component of ***ξ*** = 2^−1^*n*^−1/2^X^*T*^1 using the distributional representation in (52). Let **X** = (**x**_1_, ⋯ , **x**_p_) with each $x_{j}\in {\mathbb{R}}^{n}$. Then we have

(53)
$$\xi_{j}\;=\;2^{-1}n^{-1/2}x_{j}^{T}1\overset{d}{=}2^{-1}\left(n^{1/2}/{\left\|{\tilde{x}}_{j}\right\|}_{2}\right)n^{-1/2}{\tilde{x}}_{j}^{T}1,$$

where ${\tilde{x}}_{j}\sim N\left(0,4^{-1}I_{n}\right)$. It follows from (53) and the concentration phenomenon of Gaussian measures that each *ξ*_*j*_ is asymptotically close to *N*(0, 4^−1^) and thus consequently ***ξ*** is asymptotically close to *N*(**0**, 4^−1^ *I*_*p*_). *A key fact (i) for the finite-sample distribution of* ***ξ*** *is that the standard deviation of each component ξ*_*j*_ *converges to* 1/2 *at rate Op*(*n*^−1/2^) *that does not depend upon the dimensionality p at all*.

We now turn our attention to the second term ***η*** defined in (51). In view of (49) and the fact that *n*^−1/2^**X** is uniformly distributed on the Stiefel manifold $V_{p}\left({\mathbb{R}}^{n}\right)$, we can show that with significant probability,

(54)
$${\left\|X\hat{\beta }\right\|}_{\infty }\;\leq \;o(1)$$

for $p\sim n^{\alpha_{0}}$ with *α*_0_ < 1. The uniform bound in (54) enables us to apply the mean value theorem for the vector-valued function ***η*** around ***β***_0_ = **0**, which results in

(55)
$$\eta \;=\;n^{-1/2}X^{T}[\mu (X\hat{\beta })\;-\;\mu (0)]\;=\;4^{-1}n^{-1/2}X^{T}X\hat{\beta }\;+\;r\;=\;4^{-1}n^{1/2}\hat{\beta }\;+\;r$$

since *n*^−1/2^**X** is assumed to be orthonormal, where

(56)
$$r\;=\;n^{-1/2}X^{T}\left\{\int_{0}^{1}\left[\Sigma (tX\hat{\beta })\;-\;4^{-1}I_{n}\right]\;dt\right\}X\hat{\beta }.$$

Here, the remainder term $r\;=\;{\left(r_{1},\;\cdots \;,r_{p}\right)}^{T}\in {\mathbb{R}}^{p}$ is stochastic and each component *r*_*j*_ is generally of order *O*_*p*_{*p*^1/2^*n*^−1/2^} in light of (49) when the true model may deviate from the global null case of ***β***_0_ = **0**.

Since our focus in this theorem is the logistic regression model under the global null, we can in fact claim that each component *r*_*j*_ is generally of order *O*_*p*_{*pn*^−1^}, which is a better rate of convergence than the one mentioned above thanks to the assumption of ***β***_0_ = **0**. To prove this claim, note that the variance function *b*″(·) is symmetric in θ∈ℝ and takes the maximum value 1/4 at *θ* = 0. Thus in view of (54), we can show that with significant probability,

(57)
$$4^{-1}\;I_{n}\;-\;\Sigma (tX\hat{\beta })\;\geq \;c\text{diag}\{(tX\hat{\beta })\circ (tX\hat{\beta })\}\;=\;ct^{2}\;\text{diag}\{(X\hat{\beta })\circ (X\hat{\beta })\}$$

for all *t* ∈ [0, 1], where *c* > 0 is some constant and ≥ stands for the inequality for positive semidefinite matrices. Moreover, it follows from (49) and the fact that *n*^−1/2^**X** is uniformly distributed on the Stiefel manifold $V_{p}\left({\mathbb{R}}^{n}\right)$ that with significant probability, all the *n* components of $X\hat{\beta }$ are concentrated in the order of *p*^1/2^*n*^−1/2^. This result along with (57) and the fact that *n*^−1^**X**^*T*^**X** = *I*_*p*_ entails that with significant probability,

(58)
$$n^{-1/2}X^{T}\left\{\int_{0}^{1}\left[4^{-1}I_{n}-\Sigma (tX\hat{\beta })\right]\;dt\right\}X\;\geq \;n^{-1/2}X^{T}\left\{\int_{0}^{1}c_{*}t^{2}pn^{-1}dt\right\}X\;=\;3^{-1}c_{*}pn^{-3/2}X^{T}X\;=\;3^{-1}c_{*}pn^{-1/2}I_{p},$$

where *c*_***_ > 0 is some constant. Thus combining (56), (58), and (49) proves the above claim.

We make two important observations about the remainder term **r** in (55). First, **r** has a spherical distribution on ${\mathbb{R}}^{p}$. This is because by (55) and (51) it holds that

$$r\;=\;\eta -4^{-1}n^{1/2}\hat{\beta }\;=\;\xi \;-\;4^{-1}n^{1/2}\hat{\beta },$$

which has a spherical distribution on ${\mathbb{R}}^{p}$. Thus the joint distribution of **r** is determined completely by the marginal distribution of **r**. Second, for the nonlinear setting of logistic regression model, the appearance of the remainder term **r** in (55) is due solely to *the nonlinearity of the mean function* ***μ***(·), and we have shown that each component *r*_*j*_ can indeed achieve the worst-case order *pn*^−1^ in probability. For each 1 ≤ *j* ≤ *p*, denote by *η*_*j*_ the *j*th component of ***η***. Then in view of (49) and (55), *a key fact (ii) for the finite-sample distribution of* ***η*** *is that the standard deviation of each component η*_*j*_ *converges to* 1/2 *at rate O*_*P*_{*pn*^−1^} *that generally does depend upon the dimensionality p*.

Finally, we are ready to compare the two random variables ***ξ*** and ***η*** on the two sides of equation (51). Since equation (51) is a distributional identity in ${\mathbb{R}}^{p}$, naturally the square root of the sum of var*ξ*_*j*_’s and the square root of the sum of var*η*_*j*_’s are expected to converge to the common value 2^−1^*p*^1/2^ at rates that are asymptotically negligible. However, the former has rate *p*^1/2^*O*_*P*_(*n*^−1/2^) = *O*_*p*_{*p*^1/2^*n*^−1/2^}, whereas the latter has rate *p*^1/2^*O*_*P*_{*pn*^−1^} = *OP*{*p*^3/2^*n*^−1^}. A key consequence is that when $p\sim n^{\alpha_{0}}$ for some constant the former rate is $O_{P}\left\{n^{-\left(1-\alpha_{0}\right)/2}\right\}=o_{P}(1)$, while the latter rate becomes $O_{P}\left\{n^{3\alpha_{0}/2-1}\right\}$ which is now asymptotically diverging or nonvanishing. Such an intrinsic asymptotic difference is, however, prohibited by the distributional identity (51) in ${\mathbb{R}}^{p}$, which results in a contradiction. Therefore, we have now argued that assumption (A) we started with for 2/3 ≤ *α*_0_ < 1 must be false, that is, the asymptotic normality (11) which has been proved to hold when *p* = *o*(*n*^1/2^) generally would not continue to hold when $p\sim n^{\alpha_{0}}$ with constant 2/3 ≤ *α*_0_ ≤ 1. In other words, we have proved the invalidity of the conventional GLM p-values in this regime of diverging dimensionality, which concludes the proof of Theorem 2.

### B.4. Proof of Theorem 3

By assumption, **X** ~ *N*(**0**, *I*_*n*_ ⊗ **Σ**) with covariance matrix **Σ** nonsingular. Let us first make a useful observation. For the general case of nonsingular covariance matrix **Σ**, we can introduce a change of variable by letting $\tilde{\beta }=\Sigma^{1/2}\beta$ and correspondingly $\tilde{X}=X\Sigma^{-1/2}$. Clearly, $\tilde{X}\sim N\left(0,I_{n}⊗I_{p}\right)$ and the MLE for the transformed parameter vector $\tilde{\beta }$ is exactly $\Sigma^{1/2}\hat{\beta }$, where $\hat{\beta }$ denotes the MLE under the original design matrix **X**. Thus to show the breakdown point of the conventional asymptotic normality of the MLE, it suffices to focus on the specific case of **X** ~ *N*(**0**, *I*_*n*_ ⊗ *I*_*p*_).

Hereafter we assume that **X** ~ *N*(**0**, *I*_*n*_ ⊗ *I*_*p*_) with *p* = *o*(*n*). The rest of the arguments are similar to those in the proof of Theorem 2 in Section B.3 except for some modifications needed for the case of Gaussian design. Specifically, for the case of logistic regression model under global null (that is, ***β***_0_ = **0**), the limiting distribution in (11) becomes

(59)
$$2^{-1}n^{1/2}{\hat{\beta }}_{j}\overset{D}{\to }N(0,1),$$

since *n*^−1^**X**^*T*^**X** → *I*_*p*_ almost surely in spectrum and thus $4^{-1}n{\left(A_{n}^{-1}\right)}_{jj}\to 1$ in probability as *n* → ∞. Here, we have used a claim that both the largest and smallest eigenvalues of *n*^−1^**X**^*T*^**X** converge to 1 almost surely as *n* → ∞ for the case of *p* = *o*(*n*), which can be shown by using the classical results from random matrix theory (RMT) Geman (1980); Silverstein (1985); Bai (1999).

Note that since **X** ~ *N*(0, *I*_*n*_ ⊗ *I*_*p*_), it holds that

(60)
$$n^{-1/2}XQ\;\overset{d}{=}\;n^{-1/2}X,$$

where **Q** is any fixed *p* × *p* orthogonal matrix and $\overset{d}{=}$ stands for equal in distribution. By **X** ~ *N*(0, *I*_*n*_ ⊗ *I*_*p*_), it is also easy to see that

(61)
$$\xi \;=\;n^{-1/2}X^{T}[y\;-\;\mu (0)]\;\overset{d}{=}\;2^{-1}n^{-1/2}X^{T}1,$$

where $1\;\in \;{\mathbb{R}}^{n}$ is a vector with all components being one. In view of (49) and the assumption of **X** ~ *N*(0, *I*_*n*_ ⊗ *I*_*p*_), we can show that with significant probability,

(62)
$${\left\|X\hat{\beta }\right\|}_{\infty }\;\leq \;o\left(1\right)$$

for $p\sim n^{\alpha_{0}}$ with constant *α*_0_ < 1. It holds further that with significant probability, all the *n* components of $X\hat{\beta }$ are concentrated in the order of *p*^1/2^*n*^−1/2^. This result along with (57) and the fact that *n*^−1^**X**^*T*^**X** → *I*_*p*_ almost surely in spectrum entails that with asymptotic probability one,

(63)
$$n^{-1/2}X^{T}\left\{\int_{0}^{1}\left[4^{-1}I_{n}\;-\;\Sigma (tX\hat{\beta })\right]\;dt\right\}X\;\geq \;n^{-1/2}X^{T}\left\{\int_{0}^{1}c_{*}t^{2}pn^{-1}dt\right\}X\;=3^{-1}c_{*}pn^{-3/2}X^{T}X\to 3^{-1}c_{*}pn^{-1/2}I_{p},$$

where *c*_***_ > 0 is some constant. This completes the proof of Theorem 3.
